# A scalable method for preparing Cu electrocatalysts that convert CO_2_ into C_2+_ products

**DOI:** 10.1038/s41467-020-16998-9

**Published:** 2020-07-17

**Authors:** Taehee Kim, G. Tayhas R. Palmore

**Affiliations:** 1 School of Engineering, Brown University, Providence, RI 02912 USA; 2 Photo-Electronic Hybrids Research Center, Korea Institute of Science and Technology (KIST), Seoul, 02792 Republic of Korea

**Keywords:** Atmospheric chemistry, Electrocatalysis, Chemical engineering

## Abstract

Development of efficient catalysts for selective electroreduction of CO_2_ to high-value products is essential for the deployment of carbon utilization technologies. Here we present a scalable method for preparing Cu electrocatalysts that favor CO_2_ conversion to C_2+_ products with faradaic efficiencies up to 72%. Grazing-incidence X-ray diffraction data confirms that anodic halogenation of electropolished Cu foils in aqueous solutions of KCl, KBr, or KI creates surfaces of CuCl, CuBr, or CuI, respectively. Scanning electron microscopy and energy dispersive X-ray spectroscopy studies show that significant changes to the morphology of Cu occur during anodic halogenation and subsequent oxide-formation and reduction, resulting in catalysts with a high density of defect sites but relatively low roughness. This work shows that efficient conversion of CO_2_ to C_2+_ products requires a Cu catalyst with a high density of defect sites that promote adsorption of carbon intermediates and C–C coupling reactions while minimizing roughness.

## Abstract (web summary)

Selective reduction of carbon dioxide to high-value products is key for advancing carbon capture and utilization technologies. Here the authors prepare a copper catalyst for electrocatalytic conversion of carbon dioxide to C_2+_ products with enhanced selectivity that is attributed to a high density of surface defects.

## Introduction

Combustion of fossil fuels is the leading cause of global warming due to the accumulation of CO_2_ in the atmosphere^1,2^. The electrochemical CO_2_ reduction reaction (CO_2_RR), driven by renewable energy, is a promising strategy to reduce CO_2_ accumulation. By converting CO_2_ into products of higher value, a closed-loop carbon economy begins to emerge^3–7^. To make CO_2_RR economically viable, efficient electrocatalysts with high selectivity for targeted products at scale are needed. Among the metals studied, copper is the only metal known for its intrinsic ability to convert CO_2_ into hydrocarbons and alcohols via electrochemical CO_2_RR^3,8^. Recent progress at improving the performance of Cu-based electrocatalysts for CO_2_RR has been demonstrated with porous Cu foams^9^, Cu nanoparticle ensembles^10^, oxide-derived Cu^11^, plasma-treated Cu^12,13^, Cu nanocubes^13–15^, Cu nanowires^16–18^, wet-oxidation processed CuCl-derived Cu^19^, and single-crystal Cu^20,21^.

Despite this progress, further advances are needed for producing electrocatalysts at scale that efficiently convert CO_2_ into multi-carbon products. As such, the design of catalysts that selectively produce C_2+_ products via electrochemical CO_2_RR should focus on minimizing two competing reaction pathways: (1) the hydrogen evolution reaction (HER) and (2) C_1_ product formation (e.g., CH_4_, HCOOH). Both pathways reduce the faradaic efficiency (FE) of C_2+_ products by consuming electrons and protons. Moreover, C_1_ production reduces the amount of adsorbed carbon intermediates available for surface C–C coupling reactions.

To minimize HER, the first step of electrochemical CO_2_RR must be enhanced. This first step involves a one electron, one proton reduction of CO_2_ to form adsorbed COOH (*COOH) and is a reaction that is affected by the concentration ratio of dissolved CO_2_ to protons ([CO_2_]/[H^+^]) near the electrode surface. The relative concentration of dissolved CO_2_ and protons near the electrode surface under conditions used for the electrochemical CO_2_RR has been simulated by Gupta et al. and found to be significantly different from bulk concentrations^22^. In addition, Raciti et al. showed that high current densities observed at highly roughened electrocatalysts cause the pH to increase rapidly^17^. Despite the low concentration of protons at high pH, the efficiency of electrochemical CO_2_RR is reduced by the limited availability of dissolved CO_2_ on a rough electrode surface. Therefore, to favor electrochemical CO_2_RR over HER, a catalyst with minimal roughness should be used to mitigate a rise in local pH.

To minimize C_1_ product formation, insights gained from simulations of electrochemical CO_2_RR should guide catalyst design^23–27^. These simulations provide an energy landscape that relates the energetics of competing reactions on Cu. For example, the onset potential to form adsorbed CO (*CO) is predicted to be lowest on Cu (211) among the Cu facets simulated: (111), (100), and (211)^25^. Sandberg et al. showed that once *CO forms on a Cu surface, the barrier to forming the C–C coupling product, *OCCO, is lowest on Cu (100) relative to (111) and (211). In addition, the barrier for CO dimerization decreases with increasing *CO coverage^27^. Moreover, C_2_ product formation follows second-order kinetics with a rate proportional to the concentration of reactive C_1_ intermediates such as *CO. Here, the rate determining step is dimerization of *CO to form C_2_ products^28–30^. Other unsaturated C_1_ intermediates derived by reduction of *CO (i.e., *CHO, *COH, *CH_2_, and *CHOH) react with *CO to yield C_2+_ products^23,24,26,31,32^. Thus, high surface coverage of reactive C_1_ intermediates is essential and only obtained by a high density of surface defects. Defect sites such as grain boundaries, step sites, and vacancies that result in under-coordinated atoms on the surface of Cu are known to promote C–C coupling^33,34^. In addition, Cu^+^ and subsurface oxygen are proposed to promote the adsorption of CO_2_ and C–C coupling^12,35–38^, although the stability of subsurface oxygen remains controversial^34,35,39,40^.

To maximize the amount of Cu (100) surface, Chen et al. reported cubic microstructures of Cu formed when a Cu foil was cycled between oxidizing and reducing potentials in 0.1 M KCl^41^. Other researchers employed a similar method to prepare Cu catalysts with cubic microstructure and studied product selectivity as a function of plasma-treatment^13^, other halide ions^42^, or stability of residual oxides (indicated by ^18^O labeling)^34^. In all cases, the catalysts were more selective for ethylene than methane. However, the FE for C_2_H_4_ reported in these studies ranged between 15 and 45%. This difference is likely due to the chemical complexity of the electrochemical cycling method employed. In our hands, the electrochemical cycling method used in previous studies produces only cubic microstructures at oxidizing potentials (Supplementary Fig. 1).

**Fig. 1:**
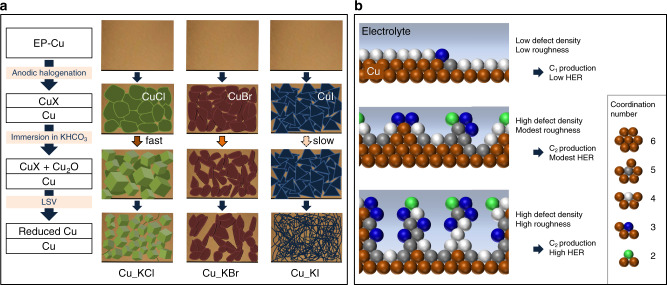
Schematics showing morphology of Cu catalysts. **a** The method used to prepare Cu catalysts for electrochemical CO_2_RR: anodic halogenation in KX solutions where X = halogen, oxide formation in basic KHCO_3_, and electroreduction in neutral KHCO_3_ by LSV. EP-Cu corresponds to electropolished Cu foils, LSV corresponds to linear sweep voltammetry. **b** Two-dimensional visualization of under-coordinated Cu atoms and surface roughness with the corresponding outcomes when used for the electrochemical CO_2_RR. This schematic is simplified but face-centered cubic Cu has a coordination number of 12.

Advances made in the previous studies inspired us to study each parameter influencing catalyst performance separately (i.e., chemical species present and their reactivity/solubility, applied potential, pH, and roughness) in order to develop a novel electrochemical method that utilizes these parameters to produce a Cu electrocatalyst selective for C_2+_ products. In this study, electrocatalysts with a balance of high density of defect sites (i.e., under-coordinated Cu) and low roughness are shown to efficiently convert CO_2_ to C_2_ and C_3_ products (FE_C2+_ of 72%) via electrochemical CO_2_RR. The electrochemical method used to produce these electrocatalysts consists of three steps: (i) anodic halogenation of Cu foils, (ii) subsequent oxide formation in a KHCO_3_ electrolyte, and (iii) electroreduction (Fig. 1). Here, chlorinated Cu, brominated Cu, or iodinated Cu were prepared by applying an oxidative potential to Cu foils immersed in 0.1 M KCl, KBr, or KI, respectively, and are henceforth denoted as Cu_KCl, Cu_KBr, and Cu_KI to reflect the different electrolytes used for anodic halogenation. Grazing-Incidence X-ray diffraction (GI-XRD) confirms the presence of Cu(I) halide on the surface of the halogenated Cu foils. Analysis of the morphological and chemical changes by SEM and EDS elucidated the processes by which subsequent surface reconstruction occurs. EDS also provides evidence that subsurface oxygen at the defect sites of Cu_KCl, Cu_KBr, and Cu_KI are produced via oxidation of Cu by high local pH of the electrolyte. The performance of these electrocatalysts provides strong evidence that both a high density of defect sites and low roughness are critical to promoting the formation of C_2+_ products via electrochemical CO_2_RR by minimizing competing HER and C_1_ production.

## Results

### Preparation of electrocatalysts

Halogenated Cu foils were prepared by applying an oxidative potential to electropolished Cu foils immersed in an electrolyte containing halide ions. A three-electrode configuration was used: Cu-foil working electrode, Pt gauze counter electrode, and Ag/AgCl reference electrode. The open circuit potential (OCP) of Cu foil immersed in 0.1 M KCl, KBr, or KI aqueous electrolyte was −0.115, −0.134, and −0.315 V vs. Ag/AgCl, respectively (see Supplementary Fig. 2). Chronoamperometric potentials of 1.1, 0.18, and −0.2 V vs. Ag/AgCl were applied to a Cu-foil working electrode while immersed in 0.1 M KCl, KBr, or KI, respectively. Note that the working electrode experiences an effective potential (*V*_eff_) defined as *V*_eff_ = *V*_app_ – *V*_oc_, where *V*_app_ is the applied potential and *V*_oc_ is the measured OCP. For example, an applied potential of −0.2 V vs. Ag/AgCl in 0.1 M KI corresponds to an effective potential of 0.115 V vs. Ag/AgCl, which anodically iodinates the Cu. Current density vs. anodic halogenation time for each electrolyte is shown in Supplementary Fig. 2.

### Evaluation of changes in the crystal structure of Cu_KX

**Fig. 2:**
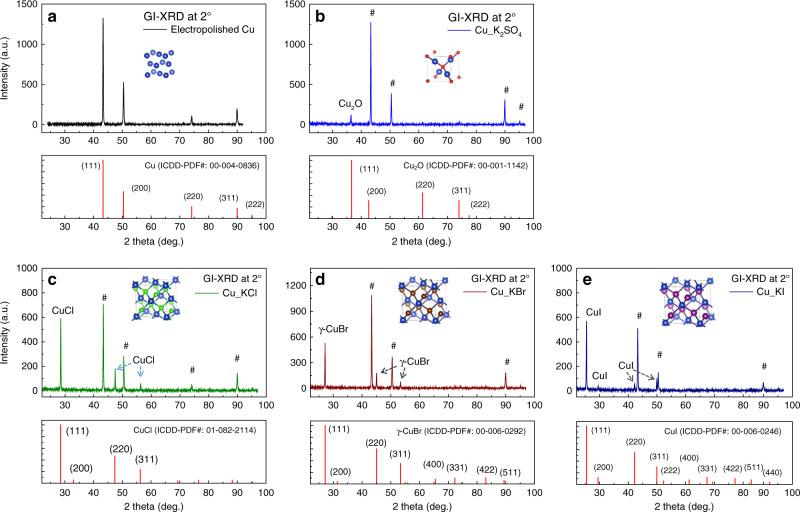
Crystal structures of halogenated Cu identified by grazing incident X-ray diffraction. GI-XRD patterns of the original electropolished Cu foil (**a**). Cu foils oxidized in 0.05 M K_2_SO_4_ at 1.1 V vs. Ag/AgCl for 300 s (control sample) (**b**), or halogenated in KCl (**c**), in KBr (**d**), and in KI (**e**) for 100, 60, and 30 s, respectively. The symbol # indicates peaks corresponding to the underlying Cu-foil substrate. The incidence angle used for GI-XRD was 2°. The inset of each GI-XRD plot is a diagram of the unit cell for Cu, Cu_2_O, CuCl, CuBr, and CuI, from (**a**) to (**e**), respectively. Cu atoms are represented as blue spheres. Source data are provided as a Source data file.

The crystal structure of Cu foils subjected to anodic halogenation was identified by grazing incident X-ray diffraction (GI-XRD) (Fig. 2). Included in this figure is GI-XRD data of control samples: the original electropolished Cu foil (Fig. 2a) and of electropolished Cu foil after being oxidized in the absence of halide ions (0.05 M K_2_SO_4_ aqueous electrolyte, 1.1 V vs. Ag/AgCl, 300 s) (Fig. 2b). The GI-XRD data of the control sample shows that oxidation in the absence of halide ions produces Cu_2_O on the surface of the Cu foil. In contrast, anodic halogenation of electropolished Cu foils in KCl, KBr, or KI results in the formation of CuCl, CuBr, or CuI, respectively (Fig. 2c–e).

### Evaluation of changes in the morphology of Cu_KX

**Fig. 3:**
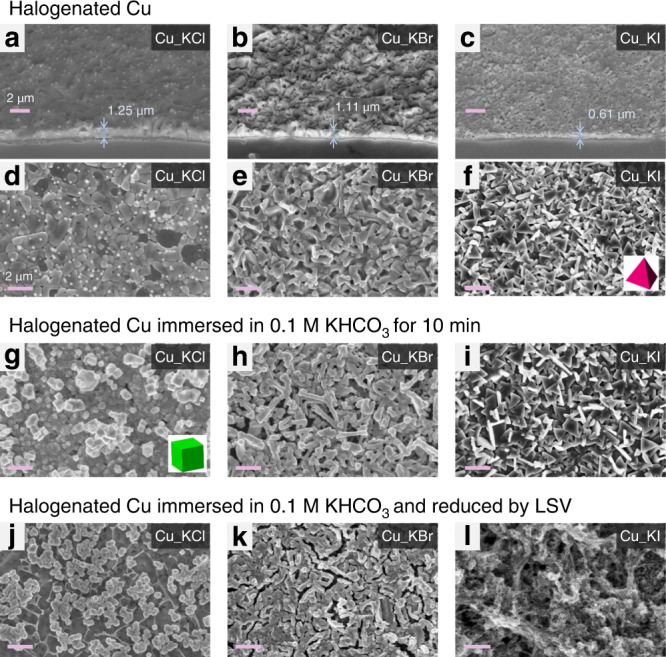
Morphology of halogenated Cu-foil electrodes. SEM images of halogenated Cu foils: **a**–**f** as-prepared; **g**–**i** after immersion in 0.1 M KHCO_3_ for 10 min; **j**–**l** after reduction by LSV in the potential range from OCP to −1.8 V in CO_2_-saturated 0.1 M KHCO_3_. **a**–**c** Cross-sectional images taken at a tilt angle of 45° to reveal the thickness of halogenated Cu. **d**–**l** Plan-view images. Anodic halogenation of Cu was performed for 50 s in 0.1 M KCl at 1.1 V (**a**, **d**, **g**, **j**); in 0.1 M KBr at 0.18 V (**b**, **e**, **h**, **k**); in 0.1 M KI at −0.2 V (**c**, **f**, **i**, **l**). All voltages are reported vs. Ag/AgCl. Scale bars: 2 μm.

The morphology of the Cu foils was expected to change with changes in crystal structure. Therefore, SEM was used to examine samples subjected to anodic halogenation for 50 s (Fig. 3). SEM images of samples halogenated for other lengths of time and at different applied potentials are shown in Supplementary Figs. 3–7 and SEM images of the control sample are shown in Supplementary Fig. 8. Cross-sectional images of the samples reveal that a halogenated Cu layer forms on electropolished Cu foils during anodic halogenation in KCl, KBr, or KI for 50 s to a thickness of 1.25, 1.11, and 0.61 μm, respectively (Fig. 3a–c). Plan-view SEM images of the as-prepared Cu(I) halide are shown in Fig. 3d–f. The formation of a surface layer of Cu(I) halide during anodic halogenation causes a volume expansion that results in surface wrinkling to relieve mechanical stress. This wrinkling is observed in Cu_KCl and Cu_KBr samples but not in the Cu_KI sample, where instead, triangle-based pyramids emerge.

The catalysts were subjected to two additional treatments to determine if the morphology of the surface changes further when halogenated Cu foils are immersed in an electrolyte commonly used for CO_2_RR experiments: (1) immersion in air-saturated KHCO_3_, where the pH is basic and (2) electrochemical reduction in CO_2_-saturated KHCO_3_, where the pH is nearly neutral (pH 6.8). These two experiments model the environment that catalysts encounter in preparation for the CO_2_RR but separate the effect of basic pH from that of reducing potentials at near neutral pH.

For the first experiment, all Cu_KX samples (where X is a halogen) were immersed in air-saturated 0.1 M KHCO_3_ for 10 min. An air-saturated solution of 0.1 M KHCO_3_ has a measured pH of 9.0, whose basicity is derived from a shift in equilibrium from bicarbonate ion to its weak acid (H_2_CO_3_) to produce OH^−^ ions:

1 $${\mathrm{HCO}}_{\mathrm{3}}^{\mathrm{ - }}\,\left( {{\mathrm{aq}}} \right)+ {\mathrm{ H}}_{\mathrm{2}}{\mathrm{O}}\,\left( {{\mathrm{aq}}} \right) \leftrightarrow {\mathrm{H}}_{\mathrm{2}}{\mathrm{CO}}_{\mathrm{3}}\,\left( {{\mathrm{aq}}} \right) + {\mathrm{OH}}^{\mathrm{ - }}\,\left( {{\mathrm{aq}}} \right)$$

When purged with CO_2_ (as in the case for the CO_2_RR), the KHCO_3_ electrolyte becomes more acidic (pH 6.8) because of the formation of carbonic acid:

2 $${\mathrm{CO}}_{\mathrm{2}} + {\mathrm{H}}_{\mathrm{2}}{\mathrm{O}} \, \left( {{\mathrm{aq}}} \right) \leftrightarrow {\mathrm{H}}_{\mathrm{2}}{\mathrm{CO}}_{\mathrm{3}}\,\left( {{\mathrm{aq}}} \right) \leftrightarrow {\mathrm{H}}^{\mathrm{ + }} \,\left( {{\mathrm{aq}}} \right)\\ + \,{\mathrm{HCO}}_{\mathrm{3}}^{\mathrm{ - }} \, \left( {{\mathrm{aq}}} \right) \leftrightarrow {\mathrm{2H}}^{\mathrm{ + }} \, \left( {{\mathrm{aq}}} \right){\mathrm{ + CO}}_{\mathrm{3}}^{{\mathrm{2 - }}} \, \left( {{\mathrm{aq}}} \right)$$

Based on calculated equilibrium diagrams, Cu_2_O is more stable than CuCl at pH 9 and OCP^43,44^. Thus, any morphological changes that may occur when Cu_KX is immersed in KHCO_3_ will be caused by an oxide-forming reaction that converts the Cu(I) halide into Cu_2_O:

3 $${\mathrm{2CuX}} \, \left( {\mathrm{s}} \right) + {\mathrm{ OH}}^{\mathrm{ - }} \, \left( {{\mathrm{aq}}} \right) \leftrightarrow {\mathrm{Cu}}_{\mathrm{2}}{\mathrm{O}}\left( {\mathrm{s}} \right) + {\mathrm{2X}}^{\mathrm{ - }} \, \left( {{\mathrm{aq}}} \right){\mathrm{ + H}}^{\mathrm{ + }} \, \left( {{\mathrm{aq}}} \right)$$

In addition, morphological changes should reflect the coordination affinity of copper(I) halides (CuCl < CuBr < CuI)^45,46^ and their solubility product (*K*_SP_) in aqueous solution (CuCl > CuBr > CuI)^42^. Consequently, when Cu_KCl is immersed in an air-saturated solution of 0.1 M KHCO_3_ (pH 9.0) for 10 min, the relatively unstable CuCl is converted rapidly to Cu_2_O with cubic morphology (Fig. 3g). The cubic morphology reflects the relative growth kinetics of different facets, where the direction of slowest growth corresponds to the largest facet^47^. Thus, the emergence of cubic morphology during the conversion of CuCl to Cu_2_O suggests that the chloride ions released during this reaction adsorb preferentially on the (100) facet, impeding its growth kinetics. This observation is consistent with simulations that have shown the preferential adsorption of halide ions onto the (100) facet of Cu^48^. When Cu_KBr is subjected to the same treatment, the wrinkled surface of CuBr appears only to shrink slightly from the release of bromide ions into the electrolyte during the oxide-forming reaction [Eq. (3)] (Fig. 3h). In contrast, when Cu_KI is subjected to the same treatment, the highly stable and insoluble CuI does not undergo any significant morphological change (Fig. 3i). GI-XRD data further supports the effect of halide ion on the extent to which CuX is converted into Cu_2_O in basic KHCO_3_ (see Supplementary Fig. 9). These observations are consistent with the trend in stability and solubility of Cu(I) halides and correspond to different rates of oxide formation via [Eq. (3)].

For the second experiment, all Cu foils that had been anodically halogenated and converted to oxide in air-saturated KHCO_3_ were reduced by LSV from the measured OCP to −1.8 V vs. Ag/AgCl at a scan rate of 5 mV/s (Supplementary Fig. 10). The resulting GI-XRD data (Supplementary Fig. 11) is nearly identical to that of the original electropolished Cu, indicating electroreduction by LSV extracts halide ions from the Cu_KX samples. Consequently, reduction of Cu_KCl results in a morphology with smaller but more uniformly sized cubic structures than before (Fig. 3j) and reduction of Cu_KBr results in further shrinkage and consequential formation of cracks (Fig. 3k). The reduction of Cu_KI results in a dramatic change to its morphology (Fig. 3i) and is attributed to the rapid reduction of iodinated Cu:

4 $${\mathrm{CuI}} \, \left( {\mathrm{s}} \right) + {\mathrm{ e}}^{\mathrm{ - }} \to {\mathrm{Cu}} \, \left( {\mathrm{s}} \right) + {\mathrm{I}}^{\mathrm{ - }} \, \left( {{\mathrm{aq}}} \right)$$

Recall, Cu_KI does not undergo significant oxide formation in the prior experiment (i.e., immersion in air-saturated KHCO_3_ electrolyte). Thus, the electrochemical reduction of Cu_KI causes an abrupt release of iodide ions, leading to the dramatic change in morphology that is observed. In contrast, bromide ions from Cu_KBr are released gradually by the oxide-forming reaction before the sample is subjected to electrochemical reduction. Cu_KCl undergoes relatively rapid oxide formation in KHCO_3_ electrolyte so that its morphology has already changed prior to being subjected to electrochemical reduction.

### Evaluation of changes in the chemical composition of Cu_KX

**Fig. 4:**
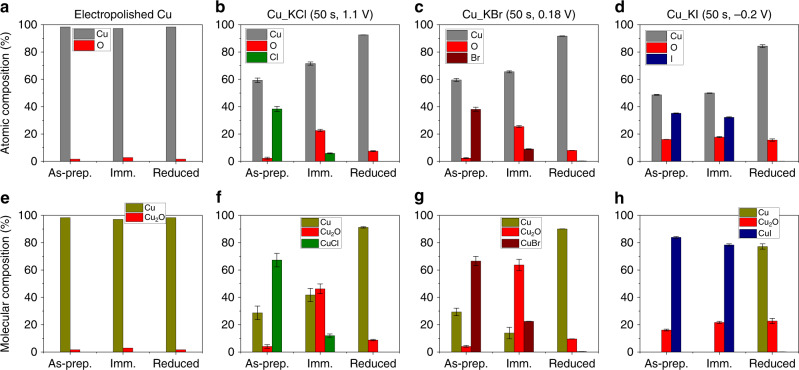
Energy-dispersive X-ray spectroscopy. **a**–**d** Raw data of atomic composition of electropolished Cu foil and halogenated Cu foils as measured by EDS. **e**–**h** Compositions of molecular species from converted EDS data. ‘As-prep.’ indicates the as-prepared catalyst by electropolishing or anodic halogenation. ‘Imm.’ indicates the catalyst underwent oxide formation by immersion in air-saturated 0.1 M KHCO_3_ electrolyte for 10 min. ‘Reduced’ indicates the catalyst was reduced in CO_2_-saturated 0.1 M KHCO_3_ electrolyte by LSV from its corresponding OCP to −1.8 V vs Ag/AgCl at a scan rate of 5 mV/s. Source data are provided as a Source data file.

The elemental compositions (Cu, O, and halogen atoms) of the surface of Cu_KX were determined using EDS for the purpose of relating changes in chemical composition to changes in morphology. Raw EDS data are shown in Fig. 4a–d, which can be converted into compositions of molecular species (Fig. 4e–h) using the chemical species identified by GI-XRD experiments. Details on the conversion of EDS data into chemical species can be found in the Supplementary Information. It is assumed that the halogenated catalysts consist of only three chemical species (i.e., Cu, Cu_2_O, and CuX) because other species such as CuO were not observed in the GI-XRD data (see Supplementary Figs. 9 and 11). In the case of electropolished Cu, only two chemical species are assumed to exist: Cu and Cu_2_O.

The initial surface species of Cu_KCl and Cu_KBr are converted into Cu_2_O by the oxide-forming reaction [Eq. (3)] when the samples are immersed in air-saturated 0.1 M KHCO_3_ for 10 min (Fig. 4f, g). For example, EDS data indicates the as-prepared Cu_KCl contains 4.06% of Cu_2_O and 67.3% of CuCl. Similarly, as-prepared Cu_KBr contains 4.13% of Cu_2_O and 66.6% of CuBr. After immersion, rapid oxide formation occurs: Cu_KCl contains 46.2% of Cu_2_O and 12.0% of CuCl while Cu_KBr contains 63.7% of Cu_2_O and 22.4% of CuBr. Unlike Cu_KCl and Cu_KBr samples, Cu_KI showed only slight changes to the composition of the initial surface species due to the high stability of CuI in basic KHCO_3_. The as-prepared sample contained 16.1% of Cu_2_O and 83.9% of CuI whereas the immersed sample contained 21.6% of Cu_2_O and 78.3% of CuI (Fig. 4h).

Electrochemical reduction of Cu_KX samples by LSV is expected to reduce all Cu(I) species to Cu^0^. The converted EDS data reveal (Fig. 4g, h), however, that 0.33% of CuBr and 0.12% of CuI remains on the surface of the respective catalysts with the Cu_KI sample having a relatively higher content of Cu_2_O (22.7%) than either the Cu_KCl or Cu_KBr samples (<10%). More detailed analysis of residual halide using XPS and EDS are shown in Supplementary Figs. 12 and 13. After electrochemical reduction, the high content of Cu_2_O in the Cu_KI is likely due to re-oxidation of the surface upon exposure to air during the time between sample preparation and EDS measurement (<30 min) and indicates that reduced Cu_KI is particularly susceptible to re-oxidation by air. This conclusion is consistent with the fact that Cu_KI undergoes abrupt morphological and chemical changes when electrochemically reduced by LSV, which generates a high density of under-coordinated atoms on the surface of the catalyst. Furthermore, this conclusion is supported by the work of Lum et al., where oxide-derived (OD) Cu with a high density of grain boundaries, could be re-oxidized very quickly when exposed to ambient air and moisture^34^.

The chemical composition of electropolished Cu does not change significantly when immersed in air-saturated KHCO_3_ for 10 min and subsequently electrochemically reduced by LSV as shown in the converted EDS data (Fig. 4e). The percentage of Cu_2_O at electropolished Cu, however, does increase slightly from 1.7 to 2.9% upon immersion in air-saturated KHCO_3_ for 10 min. This slight increase in Cu_2_O occurs via the oxidation reaction predicted by the Pourbaix diagram for copper^22,49^:

5 $${\mathrm{2}}\,{\mathrm{Cu}}\,\left( {\mathrm{s}} \right) + {\mathrm{OH}}^{\mathrm{ - }}\, \left( {{\mathrm{aq}}} \right) \leftrightarrow {\mathrm{Cu}}_{\mathrm{2}}{\mathrm{O}}\,\left( {\mathrm{s}} \right) + {\mathrm{2e}}^{\mathrm{ - }}\, \left( {{\mathrm{aq}}} \right) + {\mathrm{H}}^{\mathrm{ + }} \, \left( {{\mathrm{aq}}} \right)$$

As such, this reaction is likely to be a weak but important driving force that enables electrocatalysts to maintain a small amount of C^+^ and subsurface oxygen despite the highly negative potentials used for electrochemical CO_2_RR. The mechanism by which Cu^+^ species are stable to conditions used for CO_2_RR, however, remains indeterminate^12^. Nevertheless, because basic pH favors the oxidation reaction that forms Cu_2_O [Eq. (5)] (i.e., hydroxide ions are consumed and protons are released), the rate of this reaction is enhanced during the electrochemical CO_2_RR, where protons are consumed and the pH near the electrode increases significantly. Thus, when a catalyst has defect sites that are susceptible to re-oxidation (e.g., oxide-derived Cu^11^ or plasma-activated Cu^12^), the oxidation reaction [Eq. (5)] will generate C^+^ and subsurface oxygen at those defect sites where the local pH is high during the electrochemical CO_2_RR. Notably, Lum et al. found that oxygen species of oxide-derived Cu are unstable and reduced at the highly negative potential, however, the rapid re-oxidation of Cu possessing a high density of defect sites occurs merely by soaking in 0.1 aqueous KHCO_3_ electrolyte^34^. Their observation is consistent with Eq. (5) that basic pH of the electrolyte oxidizes Cu.

### Evaluating catalyst performance for electrochemical CO_2_RR

**Fig. 5:**
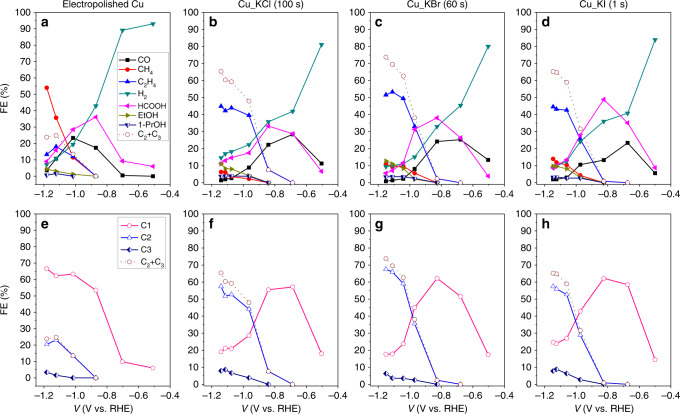
Performance of catalysts for electrochemical CO_2_ reduction reaction. Potential-dependent faradaic efficiency of the products from the CO_2_RR products on electropolished Cu (**a**), chlorinated Cu (**b**), brominated Cu (**c**), and iodinated Cu (**d**). Anodic halogenation of Cu was performed in 0.1 M KCl at 1.1 V vs. Ag/AgCl for 100 s (**b**); in 0.1 M KBr at 0.18 V vs. Ag/AgCl for 60 s (**c**); in 0.1 M KI at −0.2 V vs. Ag/AgCl for 1 s (**d**). **e**–**h** FEs of C_1_, C_2_, and C_3_ products from the same results shown in the corresponding upper plot. C_1_ products include CO, formate, and CH_4_. C_2_ products include C_2_H_4_, ethanol, acetate, acetaldehyde, and glycolaldehyde. C_3_ products include n-propanol, propionaldehyde, and allyl alcohol. Data were acquired during 40 min of electrochemical CO_2_RR at a constant potential in 0.1 M KHCO_3_ saturated with CO_2_. FE is shown as a function of the iR-corrected potential in RHE scale. Data legends apply to all plots in the same row. Source data are provided as a Source data file.

To test the activity and selectivity of the halogenated Cu catalysts, bulk electrolysis of CO_2_ was performed at a constant potential in CO_2_-saturated 0.1 M KHCO_3_ for 40 min. Electrochemical CO_2_RR experiments were performed over a potential range from −1.1 to −2.1 V vs. Ag/AgCl (with *iR*-compensation these potentials correspond to −1.1 to −1.78 V vs. Ag/AgCl or −0.50 to −1.18 V vs. RHE). The resulting potential-dependent FEs from these experiments are shown in Fig. 5. The catalysts were prepared via anodic halogenation of a Cu foil for different lengths of time (i.e., 100 s for Cu_KCl, 60 s for Cu_KBr, and 1 s for Cu_KI) to ensure complete coverage of the Cu substrate with Cu(I) halide (see Supplementary Figs. 4–6). The major product obtained on Cu(I)-halide-derived catalysts was C_2_H_4_, with its highest FE (45.1% on Cu_KCl, 49.5% on Cu_KBr, and 44.5% on Cu_KI) observed at ~ −1.15 V vs. RHE (see Table 1). For comparison, the major product obtained on electropolished Cu was CH_4_, with its highest FE (54.0%) at the same potential. Moreover, the Cu(I)-halide-derived catalysts produced CO with FEs in the range of 23–28% at potentials as low as ~ −0.68 V vs. RHE whereas electropolished Cu at this potential yielded CO with a FE of only 0.5%. Adsorbed CO (*CO) is an important intermediate required for production of C_2_ and C_3_ products via C–C bond coupling. The rate of reaction to produce C_2_H_4_ is second order with respect to the surface concentration of adsorbed CO (*CO). Thus, a high density of active sites on the surface of the catalyst is necessary to produce a high surface concentration of *CO.

**Table 1:** Representative FEs of products from electrochemical CO_2_RR shown in Figs. 5 and 6.

	Catalyst	*V*_appl_ (Ag/AgCl)	*J* (mA/cm^2^)	*V*_comp_ (Ag/AgCl)	*V*_comp_ (RHE)	FE (%)												
						CO	CH_4_	C_2_H_4_	H_2_	HCOO^−^	EtOH	PrOH	Other	Total	C_1_	C_2_	C_3_	C_2+_
Fig. 5	EP_Cu	−2.11	19.6	−1.78	−1.18	3.6	54	13.28	7.2	9.1	4.4	0.7	5.5	97.6	66.6	20.4	3.4	23.82
	Cu_KCl 100 s	−2.11	45.5	−1.73	−1.14	1.7	5.8	45.14	14.6	11.2	11	3.7	5.7	98.9	18.7	57.7	7.9	65.60
	Cu_KBr 60 s	−2.11	42.7	−1.75	−1.15	1.0	9.6	49.47	11.7	5.6	12.8	3.4	6.1	98.2	16.2	65.4	6.3	71.71
	Cu_KI 1 s	−2.11	40.0	−1.74	−1.15	2.0	14	44.54	8.9	8.7	9.8	3.2	7.7	98.8	24.7	57.4	7.8	65.21
Fig. 6	Cu_KCl 60 s	−2.11	40.3	−1.71	−1.11	1.6	4.1	50.15	12.0	9.7	10.9	4.3	5.3	98.1	15.4	62.9	7.9	70.73
	Cu_KBr 90 s	−2.11	43.3	−1.70	−1.10	2.3	1.7	50.94	13.8	8.7	12	4.4	4.2	98.0	12.6	64.4	7.2	71.54
	CI_KI 10 s	−2.11	40.0	−1.69	−1.09	1.5	8.4	49.99	9.3	9.2	13.8	5.2	3.6	98.0	19.1	65.3	7.2	72.58

**Fig. 6:**
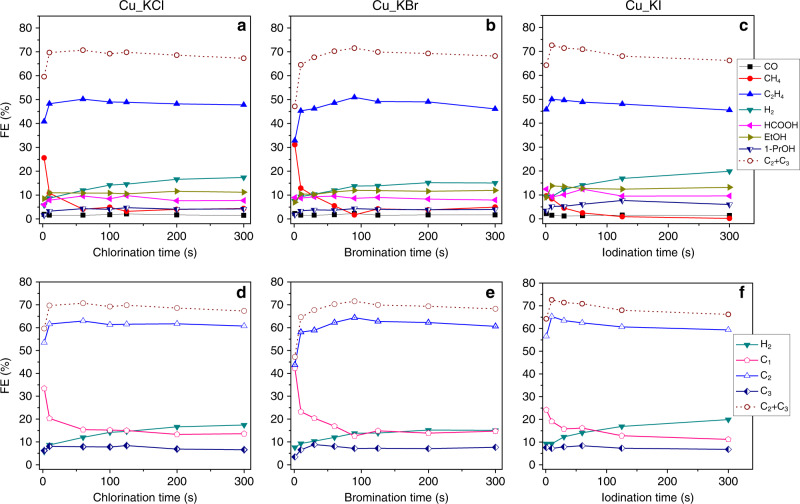
Effect of halogenation time on the performance of the catalysts for CO_2_ reduction reaction. FEs of the products from electrochemical CO_2_RR are shown versus the amount of time the Cu foils were subjected to anodic halogenation in KCl (**a**), KBr (**b**), and KI (**c**). **d**–**f** FEs of H_2_, C_1_, C_2_, and C_3_ products from the same results shown in the corresponding upper plot. Data were acquired during 40 min of electrochemical CO_2_RR at a constant potential of ~ −1.11 V vs. RHE in 0.1 M KHCO_3_ saturated with CO_2_. Data legends apply to all plots in the same row. Source data are provided as a Source data file.

The effect of anodic halogenation time on CO_2_RR performance also was investigated (Fig. 6). All data shown in Fig. 6 were collected at −1.11 V vs. RHE, where the FE for H_2_ was the lowest amongst all applied potentials studied. Supplementary Figs. 4–6 reveals that the underlying electropolished Cu substrate is exposed when anodic halogenation is performed for short periods of time. Thus, the Cu_KX catalysts produced by anodic halogenation for a short reaction time showed relatively high amounts of CH_4_ and low amounts of C_2_H_4_ and H_2_, a product distribution expected from electropolished Cu. Thus, to completely cover the underlying Cu substrate by Cu(I) halide, anodic halogenation needs to be applied for at least 60 s for Cu_KCl, 60 s for Cu_KBr and 5 s for Cu_KI. For these reaction times, 3.82, 0.80, and 0.028 C cm^−2^ of charge per unit area of electrode was used to make CuCl, CuBr, and CuI layers on electropolished Cu, respectively. The amount of charge vs. different amounts of time used for anodic halogenation is shown in Supplementary Fig. 14. A relatively short amount of time of anodic halogenation covers the Cu substrate with CuI because iodide ions have a high ligand affinity for Cu^45,46^ and the resulting CuI is highly stable^42^. In contrast, anodic chlorination or bromination of Cu requires more time to completely cover the Cu surface.

When the halogenation reaction time is increased up to 300 s, the FE for H_2_ on all three Cu(I)-halide-derived catalysts increases significantly (i.e., FE for H_2_ was 17.4% on Cu_KCl, 15.1% on Cu_KBr, and 19.9% on Cu_KI). To minimize the competing HER reaction, an optimal halogenation time was sought for each Cu_KX catalyst. The Cu_KCl catalyst that generated C_2_H_4_ with a FE of 50.2% and C_2+_ products with a FE of 70.7% was prepared using an anodic chlorination time of 60 s. Likewise, the Cu_KBr catalyst that generated C_2_H_4_ with a FE of 50.9% and C_2+_ products with a FE of 71.5% was prepared using an anodic bromination time of 90 s and the Cu_KI catalyst that generated C_2_H_4_ with a 50.0% and C_2+_ products with a FE of 72.6% was prepared using an anodic iodination time of only 10 s (see Table 1).

### Roughness factor, local pH, and competing HER

**Fig. 7:**
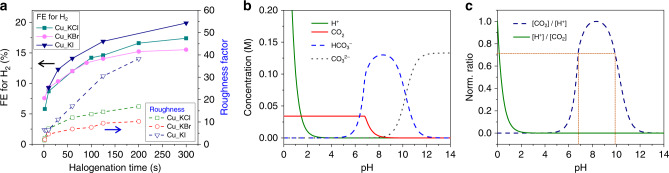
Relation between surface roughness of the electrocatalysts and HER. **a** The roughness factor of the catalyst was estimated from the double-layer capacitance as measured by cyclic voltammetry (CV) in the potential range from −0.35 to −0.5 V vs. Ag/AgCl. FE for H_2_ from Fig. 6. **b** Calculated concentrations of reactants and buffering ions in 0.1 M KHCO_3_ saturated with CO_2_. **c** The normalized concentration ratio of dissolved CO_2_ to proton as calculated from Fig. 7b. Source data are provided as a Source data file.

Anodic halogenation generates a high density of active sites, which can be crystal grain boundaries or defect sites such as step atoms or under-coordinated atoms. These active sites in turn increase the production of C_2_ products from CO_2_RR. If halogenation time is too long, however, the competing HER increases because the surface of the catalyst becomes too rough (see Supplementary Scheme 1). The roughness factor of an electrocatalyst can be determined by the double-layer (DL) capacitance method with the assumption that the surface charge is constant across different kinds of catalysts (see Supplementary Fig. 15). Figure 7a shows the relative roughness of Cu_KX as measured by DL capacitance, which are related as:

$${\mathrm{roughness}}\,{\mathrm{factor = DL}}\,{\mathrm{capacitance}}\,{\mathrm{of}}\,{\mathrm{the}}\,{\mathrm{catalyst}}\,{\mathrm{/}}\,\\ {\mathrm{DL}}\,{\mathrm{capacitance}}\,{\mathrm{of}}\,{\mathrm{the}}\,{\mathrm{electropolished}}\,{\mathrm{Cu}}$$

Higher surface roughness promotes more HER. Figure 7a shows the relationship between the roughness of the catalysts, FE for H_2_, and halogenation time. This correlation between roughness and HER can be explained by a decrease in the concentration ratio of dissolved CO_2_ to proton ([CO_2_]/[H^+^]) near the surface of the catalyst at high local pH. Although the buffering capacity of the bicarbonate electrolyte minimizes any increase in pH, the high current density observed at catalysts with high roughness rapidly depletes protons in the interfacial region and leads to a high local pH^17,22^. With high local pH, dissolved CO_2_ becomes bicarbonate and carbonate ions by the equilibrium reaction shown in Eq. (2). The concentrations of dissolved CO_2_, bicarbonate and carbonate ions, and protons in the electrolyte were calculated and are shown in Fig. 7b (details on this calculation are given in Supplementary Information and shown in Supplementary Fig. 16). The normalized concentration ratio of [CO_2_]/[H^+^] (and its inverse) is shown in Fig. 7c, which at pH 6.8 and 9.9 is 0.706. In contrast, the maximum concentration ratio of [CO_2_]/[H^+^] occurs at pH 8.3. Above pH 9.9, the concentration ratio of [CO_2_]/[H^+^] decreases rapidly so that HER is favored over electrochemical CO_2_RR. In other words, a catalyst with higher roughness produces a higher current density in electrochemical CO_2_RR. A high current density rapidly consumes reactants, which are dissolved CO_2_ and protons in the interfacial region between the catalyst and the electrolyte. The rapid consumption of protons results in a rise in the local pH. A high local pH decreases the concentration ratio of dissolved CO_2_ to proton ([CO_2_]/[H^+^]) at the interfacial region by the equilibrium reaction [Eq. (2)]. Consequently, a lower [CO_2_]/[H^+^] at a pH above 9.9 favors the competing HER side reaction. Data taken from the literature and included in Supplementary Fig. 17 also reveals a strong correlation between the roughness of the catalyst and the amount of HER. Our observation that mitigation of the rise of local pH suppresses HER is consistent with the previous report by Singh et al.^50^.

## Discussion

Hori et al. studied single-crystal Cu with a (911) facet introduced a (111) step for every five (100) basal planes [5(100) × (111)]^20^. The performance of this facet in the electrochemical CO_2_RR was superb, producing H_2_ at a FE of 12.7%, C_2_H_4_ at a FE of 50.9% and C_2+_ products at a FE of 79.5% at a stir rate of 260 rpm [in comparison to polycrystalline Cu, which in our hands produces H_2_ at a FE of 15.1%, C_2_H_4_ at a FE of 20.4% and C_2+_ products at a FE of 32.6% with a comparable rate of stirring (~290 rpm)]. However, producing single-crystal catalysts at a scale required for carbon utilization is not economically or technically feasible. Thus, a simple and inexpensive alternative to single-crystal Cu is to prepare Cu catalysts via anodic halogenation.

More recent studies have proposed the predominant facet (100) of cubic structures of Cu promotes the conversion of CO_2_ into multi-carbon products^14,19^. Although Cu_KCl exhibits cubic structures on its surface, both Cu_KBr and Cu_KI have irregular microstructures (see Fig. 3). All three Cu_KX catalysts, however, have a high density of defect sites and produce C_2+_ products with FEs up to 70.73% on Cu_KCl, 71.54% on Cu_KBr, and 72.58% on Cu_KI. Thus, in addition to specific crystal facets, a high density of defect sites (i.e., step sites and low-coordinated Cu atoms) is essential for converting CO_2_ into C_2_ products via electrochemical CO_2_RR at a Cu catalyst. This finding is consistent with the results of Hori et al. that Cu (100) single crystal is not an ideal facet for selective C_2+_ production (FE_CH4_ = 30.4% and FE_C2H4_ = 40.4%)^20^ and the more recent claim that the presence of oxygen species in surface and subsurface regions of catalysts with nano-cubic morphology are more important for achieving high activity and selectivity for C_2+_ hydrocarbons or alcohols than the presence of Cu(100) facets themselves^13^.

Anodic halogenation of electropolished Cu followed by surface reconstruction from base-induced oxide formation and electroreduction creates a surface with a high density of defect sites. These sites stabilize species such as Cu^+^ and subsurface oxygen, which are known to promote C_2+_ production during the electrochemical CO_2_RR. Evidence of a high density of defect sites on the surface of Cu_KX catalysts is provided by incidence-angle dependent GI-XRD data (Supplementary Fig. 18), which shows decreased crystal ordering at the surface of Cu_KBr during surface reconstruction. Furthermore, EDS data reveals the high susceptibility of these surfaces to re-oxidation. The density of defect sites in oxide-derived Cu has recently been determined using positron annihilation spectroscopy (PAS)^35^. Based on our results, we conclude that a high density of defect sites is the most important attribute of a Cu catalyst that selectively converts CO_2_ into C_2+_ products via the electrochemical CO_2_RR.

Interestingly, ethane (C_2_H_6_) is produced when the roughness factor exceeds 30 (FE_C2H6_ = ~1.2% in this work). The mechanistic pathway to produce C_2_H_6_ has been proposed to be the reaction between adsorbed ethylene (*C_2_H_4_) and adsorbed hydrogen (*H)^51^. Therefore, observation of C_2_H_6_ indicates high surface concentrations of both *C_2_H_4_ and *H, which only can be attributed to a high density of defect sites and high roughness, respectively. Thus, production of C_2_H_6_ indicates that the roughness of the catalyst needs to be lowered to obtain the optimal balance of a high density of defect sites that favors C_2_H_4_ production and low roughness that suppresses HER.

Recently, Wang et al. reported a wet-chemistry method to prepare cuprous-halide-derived catalysts with FE up to 58% for C_2+_ product^52^. Our study significantly advances this reaction to obtain a FE of 72.6% for C_2+_ product. This advance is made possible by the discovery that features of Cu catalysts can be optimized for this reaction (e.g., defect density, roughness) by controlling the duration of anodic halogenation at a constant potential. Our study also highlights the importance of the correlation between the roughness of the catalyst and HER and the impact of that correlation on electrochemical CO_2_RR at negative potentials (~ −1 V vs. RHE). Thus, the [CO_2_]/[H^+^] ratio provides an important parameter that can be controlled to yield a catalyst that is both efficient and selective for C_2+_ products.

In summary, Cu(I)-halide-derived catalysts were prepared using anodic halogenation. The optimal time and voltage for anodic halogenation was 60–100 s at 1.1 V, 60–90 s at 0.18 V, and 10 s at −0.2 V vs. Ag/AgCl for Cu_KCl, Cu_KBr, and Cu_KI, respectively. Iodide ions react with the Cu surface rapidly at weak oxidative potentials because of the high affinity of I^−^ to form CuI. All Cu(I)X-derived catalysts (where X = Cl, Br, or I) were found to be excellent catalysts for producing C_2+_ products via the electrochemical CO_2_RR with FE_C2+_ of 70.7%, 71.5%, and 72.6% on Cu_KCl, Cu_KBr, and Cu_KI, respectively. By exploiting volume changes that occur during anodic halogenation and subsequent surface reconstruction, we show that anodic halogenation is a simple to perform and scalable method for consistently preparing Cu catalysts with a high density of surface defect sites and low roughness. The high density of defect sites promotes production of multi-carbon products and the low roughness suppresses the competing HER. These results, taken together, provide an approach to preparing catalysts for efficient conversion of CO_2_ to C_2+_ products that has characteristics desirable for carbon utilization technologies: simple to perform, consistent, regenerative, and scalable.

## Methods

### Preparation of electrocatalysts

All Cu foils were mechanically polished with 400 grit sandpaper, and rinsed with deionized (DI) water. The Cu foils (2 × 5 cm^2^) subsequently were electropolished by chronoamperometry in 85% phosphoric acid at 1.5 V with a Cu counter electrode in a two-electrode configuration. The electropolished Cu foils were rinsed with DI water. After cutting the electropolished Cu foils into 2 × 0.5 cm^2^ pieces, the foils were flattened and both the back side and part of the front side were covered with polyimide (PI) tape to define the geometric area of the working electrode. The electrode was wrapped in PTFE tape (Swagelok) to prevent detachment of the PI tape. The exposed geometric area was typically 0.35 cm^2^. KCl (Macron fine chemicals), KBr (Fisher Scientific), and KI (Fisher Scientific) were dissolved in DI water to a concentration of 0.1 M. Anodic chlorination, bromination, and iodination was performed on an electropolished Cu foil immersed in 0.1 M KCl, KBr, and KI at 1.1, 0.18, and −0.2 V vs. Ag/AgCl, respectively, in a three-electrode configuration using a potentiostat (Pine Instrument Company, Biopotentiostat, model AFCBP1). The counter electrode was Pt gauze and the reference electrode was Ag/AgCl (saturated KCl) electrode. The OCP of electropolished Cu in 0.1 M KCl, KBr, and KI was −0.115, −0.134, and −0.315 V vs. Ag/AgCl, respectively (Supplementary Fig. S1).

### Characterization of the electrocatalysts

SEM images were acquired using a LEO 1530 VP ultra-high resolution field emitter SEM at 10 kV. Elemental analysis of samples was obtained using the EDS accessory (Oxford Instruments, Inca X-sight, model 7426) of the SEM. The GI-XRD data were obtained using a Bruker D8 Discovery High resolution X-ray Diffractometer at incidence angle of 2° and wavelength of 1.54 Å. The double-layer capacitance was measured by cyclic voltammetry in the potential range from −0.35 to −0.5 V vs. Ag/AgCl in CO_2_-saturated 0.1 M KHCO_3_ after electrochemical CO_2_RR. XPS equipment of PHI 5000 VersaProbe (Ulvac-PHI, Japan) was used to characterize the surface of the Cu catalysts.

### Electrochemical CO_2_ reduction

Electrochemical CO_2_RR was carried out in a custom made two compartment cell, separated by a Nafion 117 proton-exchange membrane. The two compartments were filled with 8.2 ml of 0.1 M KHCO_3_ (Sigma-Aldrich, ≥99.95%) electrolyte. A three-electrode configuration was employed: Cu-foil working electrode, Pt gauze counter electrode, and a home-built Ag/AgCl reference electrode. The working and reference electrodes were placed in the cathode compartment and the Pt gauze counter electrode was placed in the anode compartment. Prior to initiating electrochemical CO_2_RR, the halogenated Cu-foil electrode was immersed in 0.1 M KHCO_3_ electrolyte and linear sweep voltammetry was performed with a scan rate of 5 mV/s from the OCP to the working potential (usually −0.2 to −2.1 V vs Ag/AgCl). Subsequently, CO_2_RR was performed with fresh electrolyte saturated with CO_2_. Before and during electrochemical CO_2_RR, the cell was purged continuously with CO_2_ at a flow rate of 20 mL/min as measured with a rotameter (OMEGA FL-3841G FT-032-41-GL-VN). Electrochemical CO_2_RR was performed by chronoamperometry for 40 min with a magnetic stirring bar spinning at ~1500 rpm. A Thermolyne Nuova stir plate (model No. SP18425) was used to stir a 1-cm-long magnetic bar in the electrolyte. The stirring speed was calibrated in comparison with the Fisher Scientific hot plate/stirrer (Cat. No. 11-100-49SH). It is worth noting that all experimental results on electrochemical CO_2_RR were obtained while stirring the electrolyte with a magnetic stirrer at ~1500 rpm.

After electrochemical CO_2_RR, the solution resistance (*R*) was measured with a potentiostatic electrochemical impedance spectrometer (Solartron, 1255 HF Frequency Response Analyzer) at 10 kHz. All electrochemical data was collected vs. Ag/AgCl reference. The *iR*-compensated potentials relative to the reversible hydrogen electrode (RHE) are shown in the tables in the supporting information using the following equations:

$${\it{V}}_{{\mathrm{comp}}}\left( {{\mathrm{Ag/AgCl}}} \right)={\it{V}}_{{\mathrm{appl}}}\left( {{\mathrm{Ag/AgCl}}} \right)+{\it{iR}}$$

$${\it{V}}_{{\mathrm{comp}}}\left( {{\mathrm{RHE}}} \right)={\it{V}}_{{\mathrm{comp}}}\left( {{\mathrm{Ag/AgCl}}} \right)+0.197+0.059 \ast {\mathrm{pH}}$$

Liquid-phase products in the catholyte were collected for quantification using nuclear magnetic resonance (NMR).

### Product analysis

The reduction compartment of the gas-tight reactor was connected to the inlet of the sample loop of a gas chromatograph (GC, Buck Scientific, Model 910). GC measurements were performed on sample injections taken after 10 and 38 min of the CO_2_RR to determine the concentration of gaseous products present: CO, CH_4_, C_2_H_4_, H_2_. The GC was equipped with a methanizer and a flame ionization detector (FID) to detect CO and hydrocarbons and a thermal conductivity detector (TCD) to detect H_2_. Nitrogen was used as the carrier gas. Liquid products were quantified using 1D ^1^H NMR (400 MHz, Bruker high field NMR spectrometers). Each sample of catholyte (700 μL) was mixed with 35 μL of a D_2_O solution containing internal standards: 50 mM phenol and 10 mM dimethyl sulfoxide (DMSO). The water peak was suppressed by a WET procedure (Bruker). The acquired NMR data were processed with Topspin 4.0.5 software. The peak area of the liquid product (formate) at higher chemical shift with respect to the suppressed water peak (chemical shift = 4.7 ppm) was normalized to the peak area of phenol (chemical shift = 7.2 ppm). The peak areas of the liquid products (acetate, ethanol, propanol, acetaldehyde, propionaldehyde, glycolaldehyde, and allyl alcohol) at lower chemical shift with respect to the suppressed water peak were normalized to the peak area of DMSO (chemical shift = 2.6 ppm).

## Supplementary information

Supplementary Information

## Data Availability

The data supporting the findings of this study are available within the article and Supplementary Information file. All other relevant source data are available from the corresponding author upon request. The source data underlying Figs. 2, 4–7 and Supplementary Figs. 1, 2, 9–15, 18, and 19 are provided as a Source data file.

## Source data

Source Data
