# Supplementary material for: A scalable method for preparing Cu electrocatalysts that convert CO2 into C2+ products
Source: Nat Commun. 2020 Jul 17;11:3622. doi: 10.1038/s41467-020-16998-9 (PMC7368024; doi:10.1038/s41467-020-16998-9)
Supplement: Supplementary file 1 — Supplementary Information [file 41467_2020_16998_MOESM1_ESM.pdf]

Supplementary Information

A Scalable Method for Preparing Cu Electrocatalysts that Convert CO_2 into C_{2+} Products

Taehee Kim[1,2] & G. Tayhas R. Palmore[1]

[1] School of Engineering, Brown University, Providence, RI 02912, United States

[2] Photo-electronic Hybrids Research Center, Korea Institute of Science and Technology (KIST), Seoul 02792, Republic of Korea

Correspondence and requests for materials should be addressed to G. T. R. P.

(email: Tayhas_Palmore@brown.edu)

Supplementary Information include 20 Supplementary Figures, 8 Supplementary Table, 6 Supplementary Notes, and Supplementary References

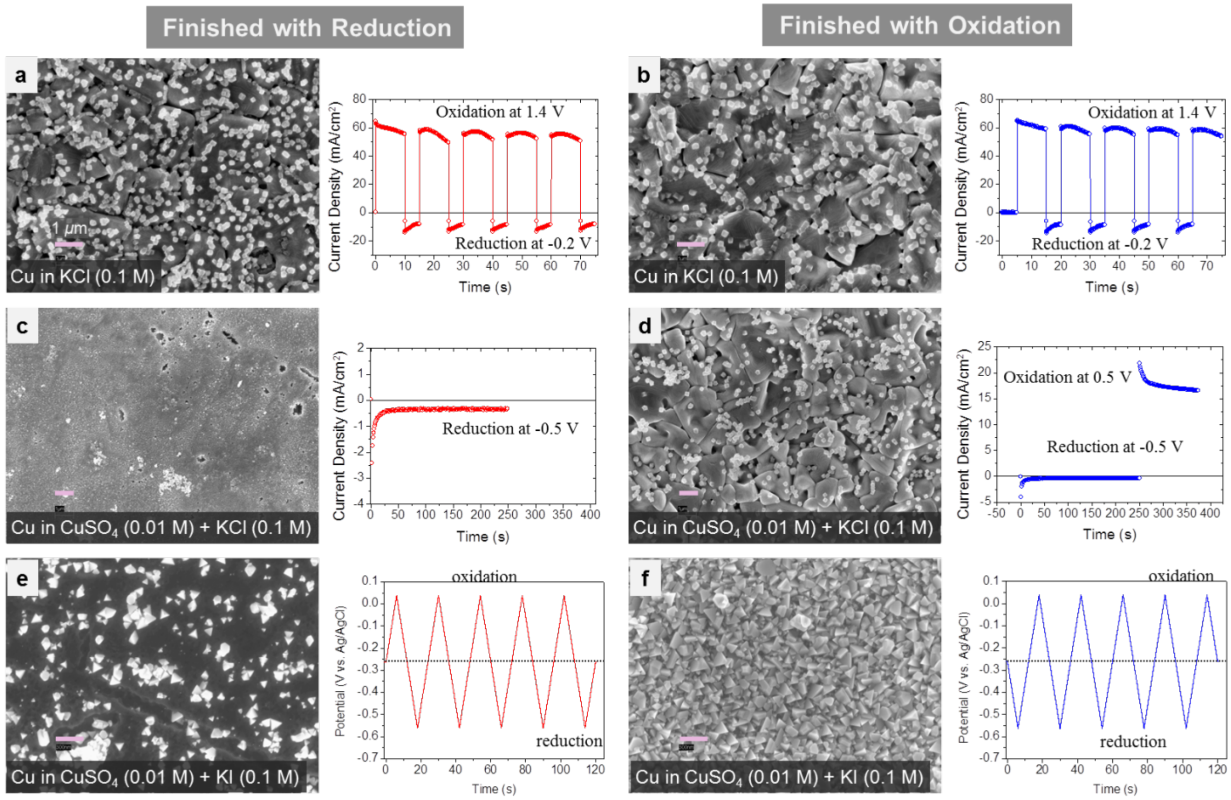

Supplementary Figure 1. Comparison of morphology of Cu foils subjected to electrochemical cycling reported by others and stopped at either reductive (left) or oxidative potential (right). SEM image of a Cu foil cycled between 1.4 V and -0.2 V in 0.1 M KCl and ending at (a) a reductive potential or (b) an oxidative potential and their corresponding current density vs. time plots. For each cycle, the potential was held at positive and negative potentials for 10 and 5 s, respectively.[1] (c) SEM image of a Cu foil after electrodeposition in 0.01 M Cu_2SO_4 + 0.1 M KCl at -0.5 V for 250 s and corresponding current density vs. time plot. (d) SEM image of a Cu foil after one cycle in 0.01 M Cu_2SO_4 + 0.1 M KCl where potential was held at -0.5 V for 250 s and subsequently held at 0.5 V for 125 s and corresponding current density vs. time plot. (e) SEM image of a Cu foil cycled between V_P and V_N in 0.01 M Cu_2SO_4 + 0.1 M KI at 50 mV/s where $V_P = V_{OC} + 0.3$ V and $V_N = V_{OC} - 0.3$ V and $V_{OC} = -0.262$ V, ending with a reductive cycle and corresponding applied potential vs. time plot. (f) SEM image of a Cu foil cycled between V_P and V_N in 0.01 M Cu_2SO_4 + 0.1 M KI at 50 mV/s, ending with an oxidative cycle and corresponding applied potential vs. time plot. All plots reflect geometric current density, potentials referenced to Ag/AgCl, and scale bars = 1 micron. Comparing data reveals similar morphologies (mesostructures of CuCl and small cubes of Cu_2O) in (a) and (b); formation of small cubic structures and mesostructures only at an oxidative potential (d) not (c); preservation of CuI structures when potential cycling ends at an oxidative potential (f) not (e). Source data are provided as a Source Data file.

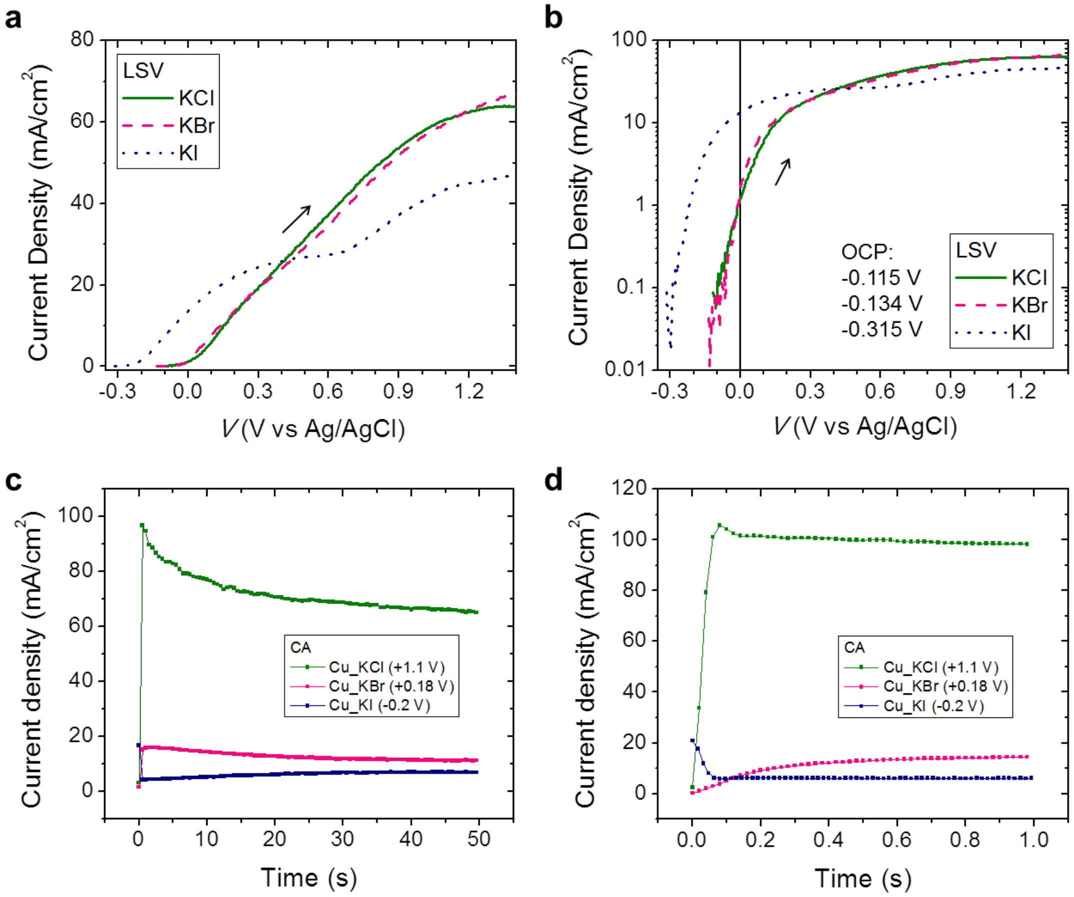

Supplementary Figure 2. Anodic halogenation of Cu foils. Linear sweep voltammograms of electropolished Cu foils in 0.1 M KCl, KBr, or KI electrolyte (a). Data in (a) replotted on a logarithm scale (b). Chronoamperograms of anodic halogenation of Cu foils for 50 s (c). Data shown in (c) zoomed in to highlight initial stage of procedure (d). The arrows in (a) and (b) indicate the sweep direction. All current densities correspond to geometric current density. Source data are provided as a Source Data file.

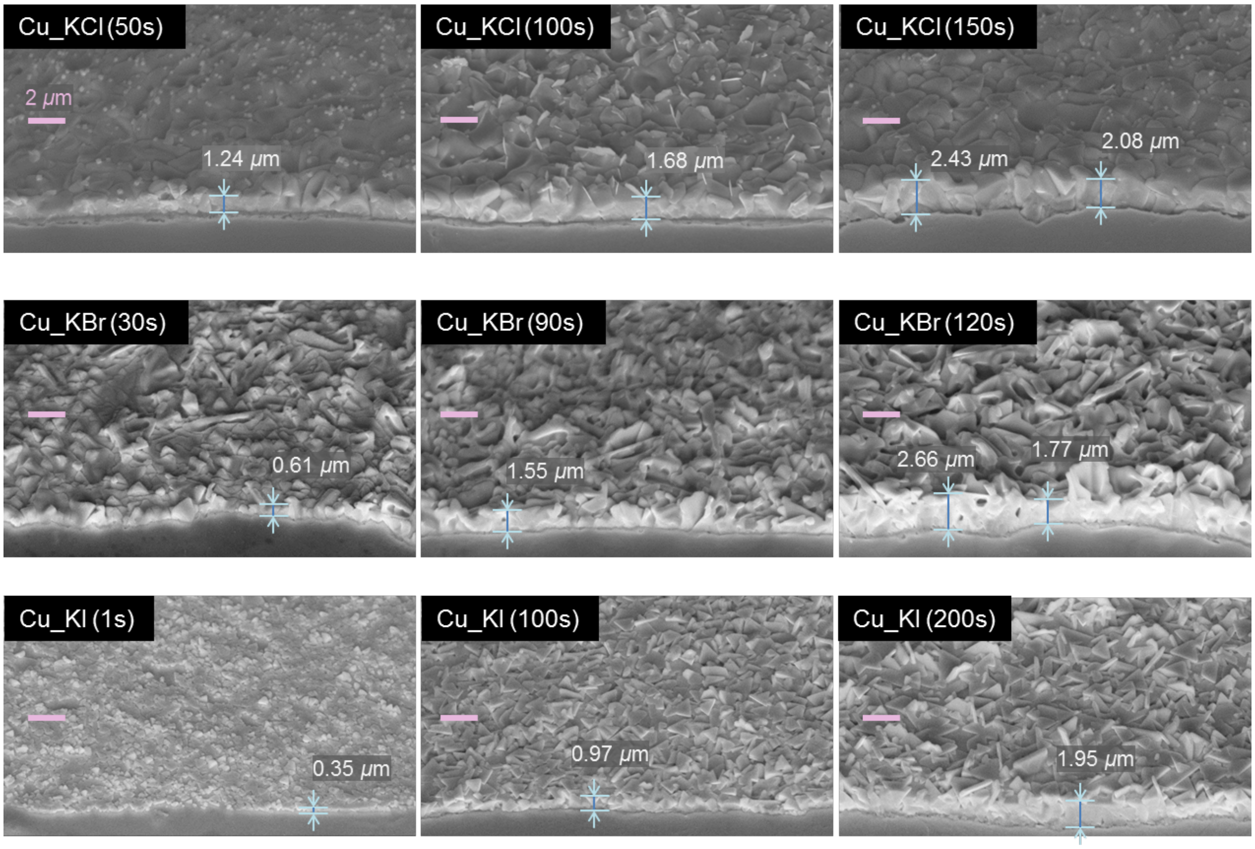

Supplementary Figure 3. Cross-sectional SEM images of Cu_KCl, Cu_KBr, and Cu_KI samples prepared by anodic halogenation for different amounts of time. Images were taken at a tilt angle of 45° and all scale bars correspond to 2 microns. These images reveal that increasing the anodic halogenation time results in an increase in the thickness of CuX.

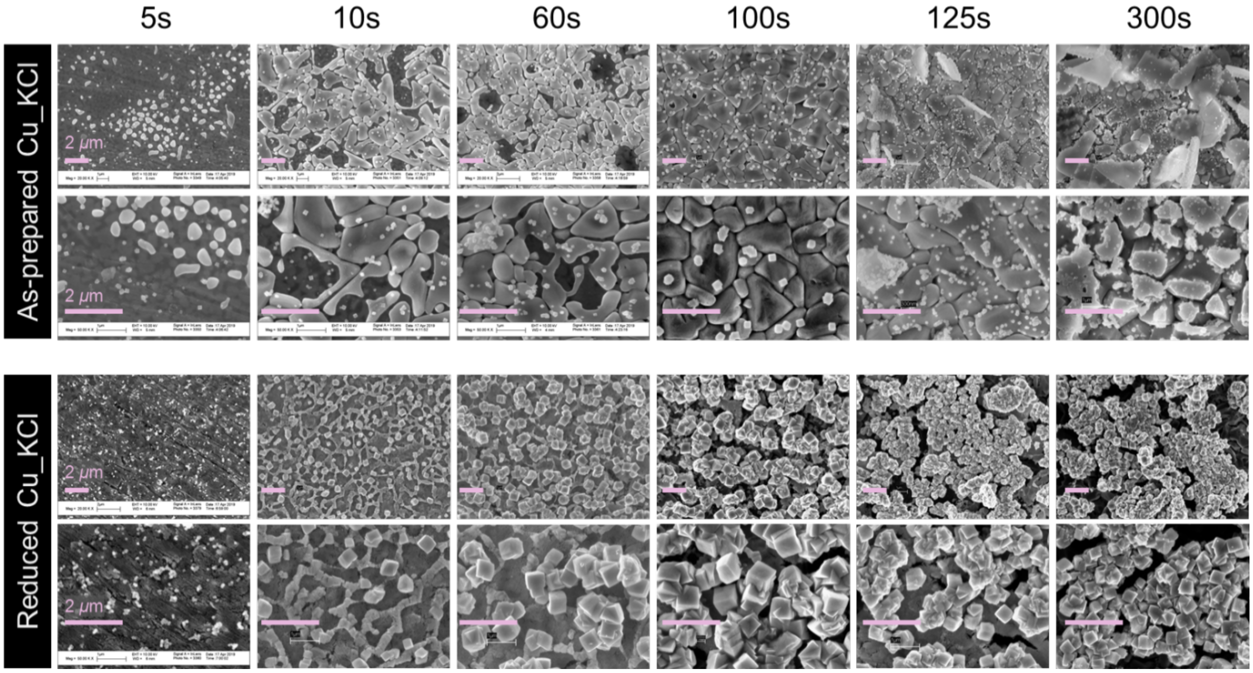

Supplementary Figure 4. Plan-view SEM images of Cu_KCl prepared by anodic halogenation for different amounts of time. Scale bars correspond to 2 microns. Samples were subsequently reduced (LSV) from the OCP to -1.8 V vs. Ag/AgCl in CO_2-saturated 0.1 M $KHCO_3$. These images reveal that anodic chlorination for less than 60s does not produce a high density of defects on all areas of exposed Cu foils.

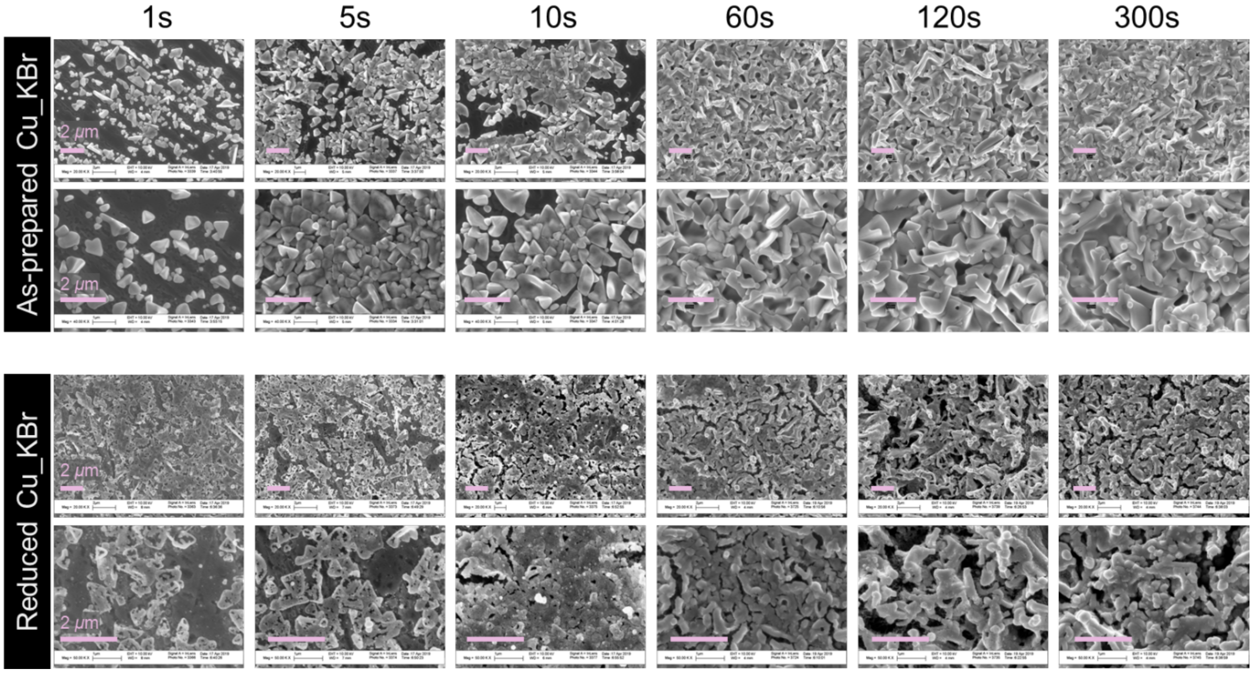

Supplementary Figure 5. Plan-view SEM images of the brominated Cu prepared by anodic halogenation for different amounts of time. Scale bars correspond to 2 microns. Samples of Cu_KBr were subsequently reduced (LSV) from the OCP to -1.8 V vs. Ag/AgCl in CO_2-saturated 0.1 M $KHCO_3$. These images reveal that at least 60s of anodic halogenation is required to cover the Cu foil completely with CuBr.

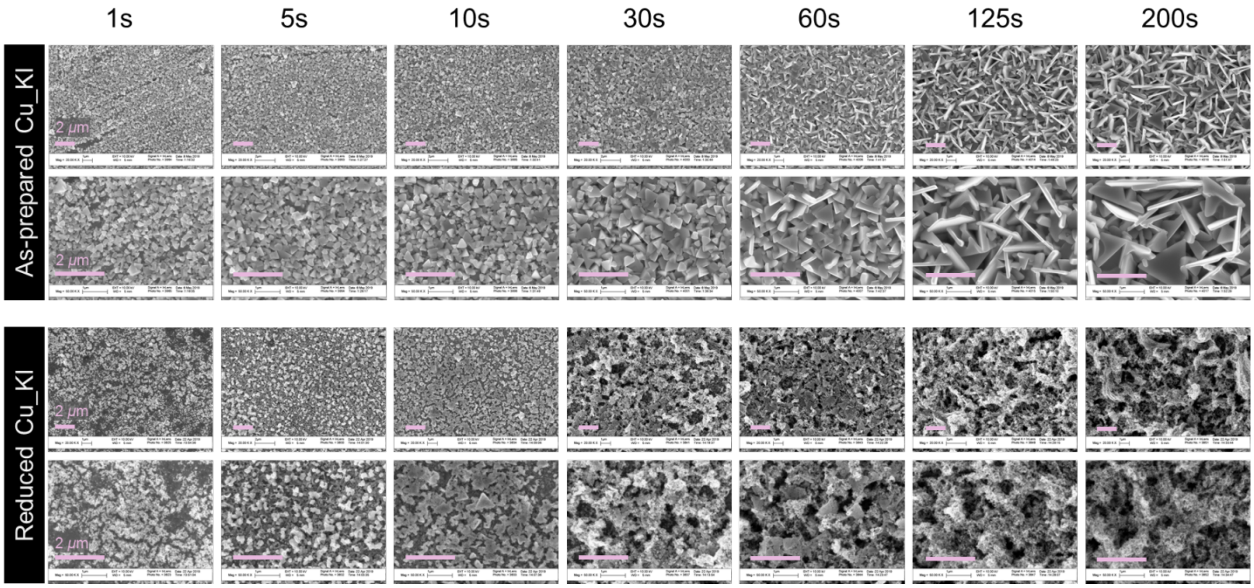

Supplementary Figure 6. Plan-view SEM images of the iodinated Cu prepared by anodic halogenation for different amounts of time. Scale bars correspond to 2 microns. Samples of Cu_KI were subsequently reduced (LSV) from the OCP to -1.8 V vs. Ag/AgCl in CO_2-saturated 0.1 M $KHCO_3$. These images reveal that 10 s of anodic iodination completely covers the underlying Cu foil with CuI and longer times of anodic iodination (i.e., 200 s) makes the surface too rough.

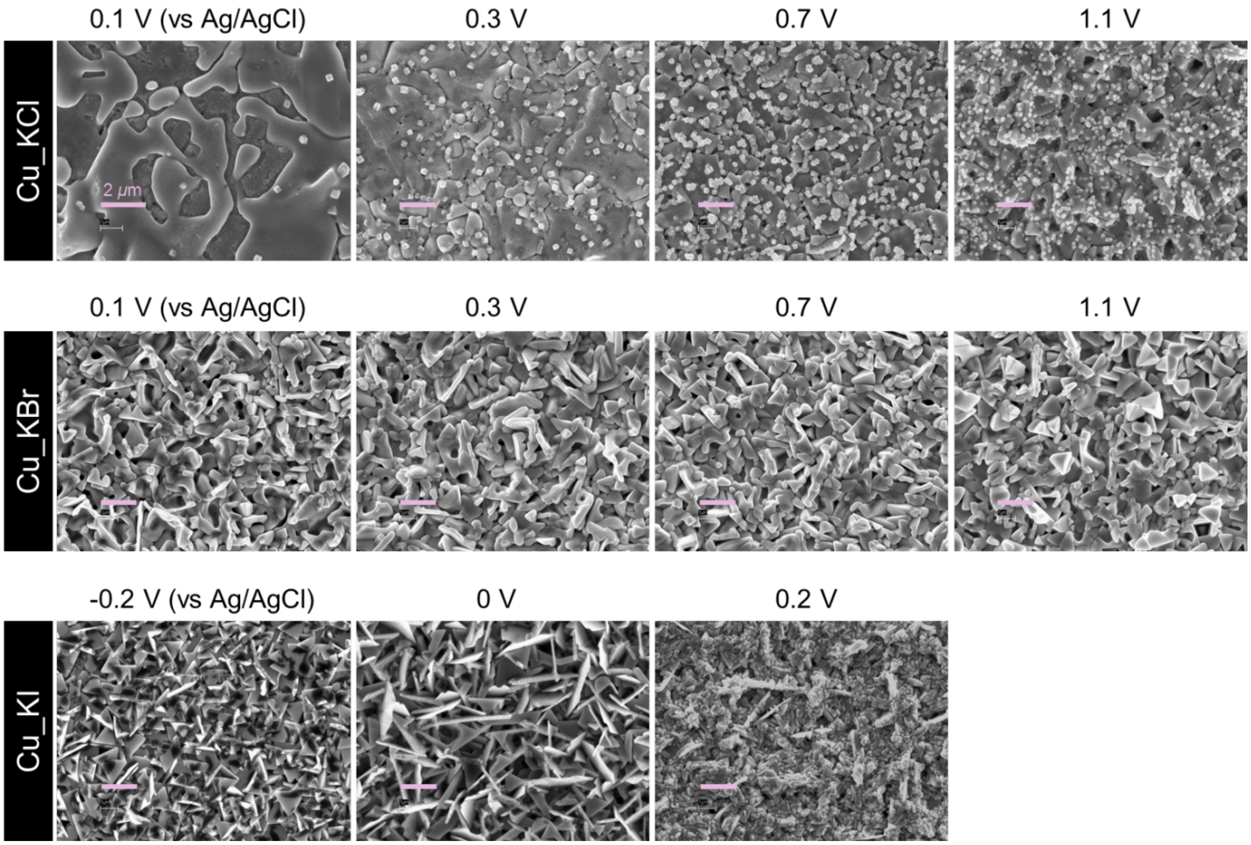

Supplementary Figure 7. Plan-view SEM images of chlorinated Cu (Cu_KCl), brominated Cu (Cu_KBr), and iodinated Cu (Cu_KI) after anodic halogenation for 125 s at different applied potentials. Scale bars correspond to 2 microns.

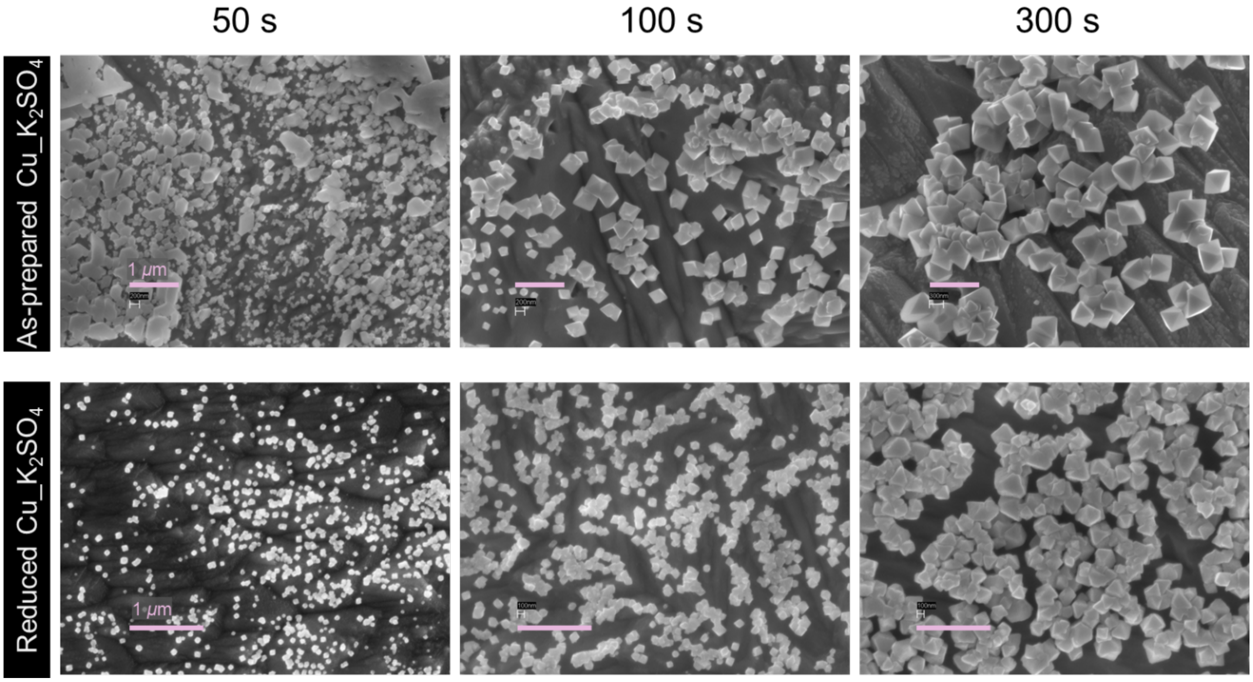

Supplementary Figure 8. Plan-view SEM images of electropolished Cu foil anodically oxidized in 0.05 M K_2SO_4 for different amounts of time and subsequently reduced by LSV from the OCP to -1.8 V vs. Ag/AgCl in CO_2-saturated 0.1 M $KHCO_3$. Scale bars correspond to 1 micron. Compared to the SEM images of Cu_KX samples, these images reveal that anodic oxidation of electropolished Cu foil in the absence of halogen ions does not result in complete coverage of the underlying substrate with an oxide layer even after 300 s of oxidation. Moreover, octahedral crystals with (111) facets develop on the surface of the Cu foil. Consequently, electrochemical CO_2 RR at these electrodes yields products (i.e., C_1 products) expected from polycrystalline Cu and Cu(111) in particular.

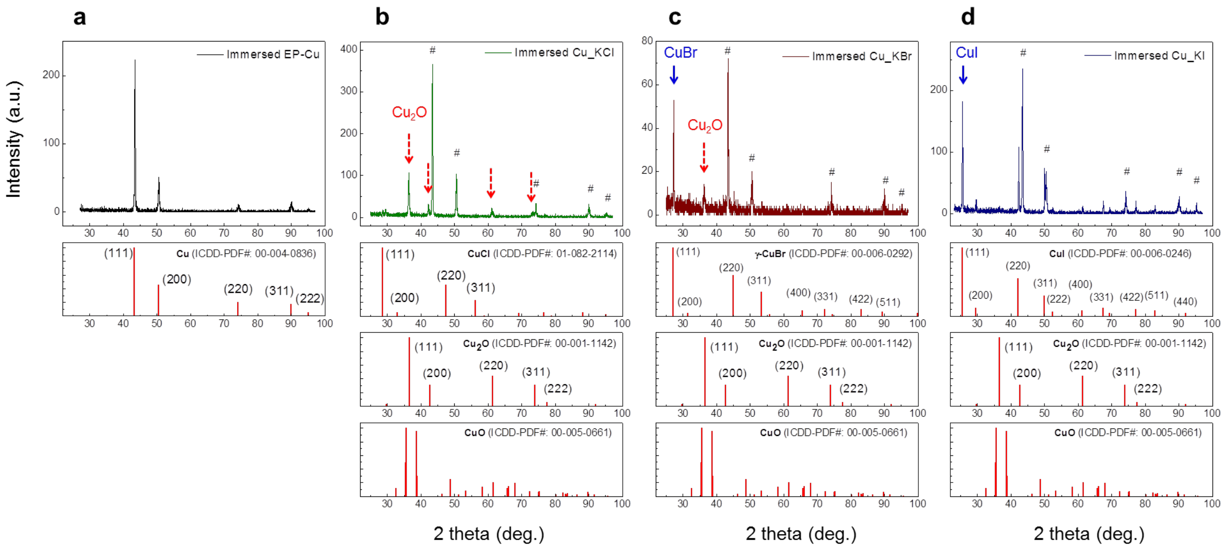

Supplementary Figure 9. GI-XRD data of the electropolished Cu foil (a), Cu_KCl (b), Cu_KBr (c), and Cu_KI (d) after immersion in air-saturated 0.1 M $KHCO_3$ for 10 min. Peaks corresponding to CuO were not observed in any of the XRD measurements. Red dashed arrows identify peaks corresponding to Cu_2O and # identify peaks corresponding to the underlying Cu foil substrate. (a) Peaks corresponding to Cu_2O are not observed. A thin layer or small domains of Cu_2O on the surface of electropolished Cu foil (as indicated by EDS) may not be enough material to produce an observable XRD peak. (b) Peaks corresponding to Cu_2O are observed and the peak corresponding to CuCl (2theta = 28.5°) decreased significantly. This result indicates that most of the CuCl is converted into Cu_2O when Cu_KCl is immersed in air-saturated $KHCO_3$. (c) Peaks corresponding to CuBr (2theta = 27.1°) and Cu_2O (2theta = 36.6°) are observed. This result indicates that CuBr is converted into Cu_2O only partially when Cu_KBr is immersed in air-saturated $KHCO_3$. (d) Peaks corresponding to Cu_2O were not observed. If defect sites on the surface and subsurface of Cu_KI forms Cu_2O by immersion in air-saturated $KHCO_3$, its crystalline domains were not large enough to produce peaks corresponding Cu_2O. Source data are provided as a Source Data file.

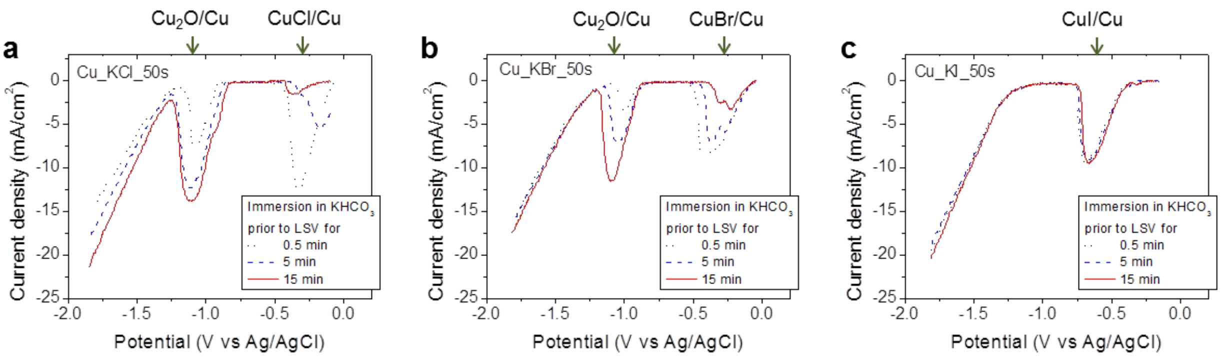

Supplementary Figure 10. Linear sweep voltammetry (LSV) data of Cu_KCl (a), Cu_KBr (b), and Cu_KI (c) after different amounts of time immersed in air-saturated 0.1 M $KHCO_3$. All samples were anodically halogenated for 50 s and subsequently immersed in 0.1 M $KHCO_3$ for 0.5 min, 5 min, or 15 min. Afterwards, the samples were reduced by LSV from the OCP to -1.8 V at a scan rate of 5 mV/s. The peak at -1.1 V vs. Ag/AgCl in the LSV data corresponds to the reduction of Cu_2O: Cu_2O (s) + 2e$^-$ (aq) + H$^+$ (aq) → 2Cu (s) + OH$^-$ (aq). The peaks at less negative potentials between -0.3 to -0.6 V vs. Ag/AgCl correspond to the reduction of CuX: CuX (s) + e$^-$ → Cu (s) + X$^-$ (aq). The LSV corresponding to Cu_KCl immersed in $KHCO_3$ for 15 min shows a significantly smaller peak at -0.3 V vs. Ag/AgCl and a larger peak at -1.1 V vs. Ag/AgCl compared to samples immersed for shorter times. This result provides evidence that CuCl was converted rapidly to Cu_2O upon immersion in air-saturated $KHCO_3$. The LSV curves corresponding to Cu_KI show almost no change regardless of time immersed in air-saturated $KHCO_3$, confirming the relative stability of CuI to basic conditions. All current densities correspond to geometric current density. Source data are provided as a Source Data file.

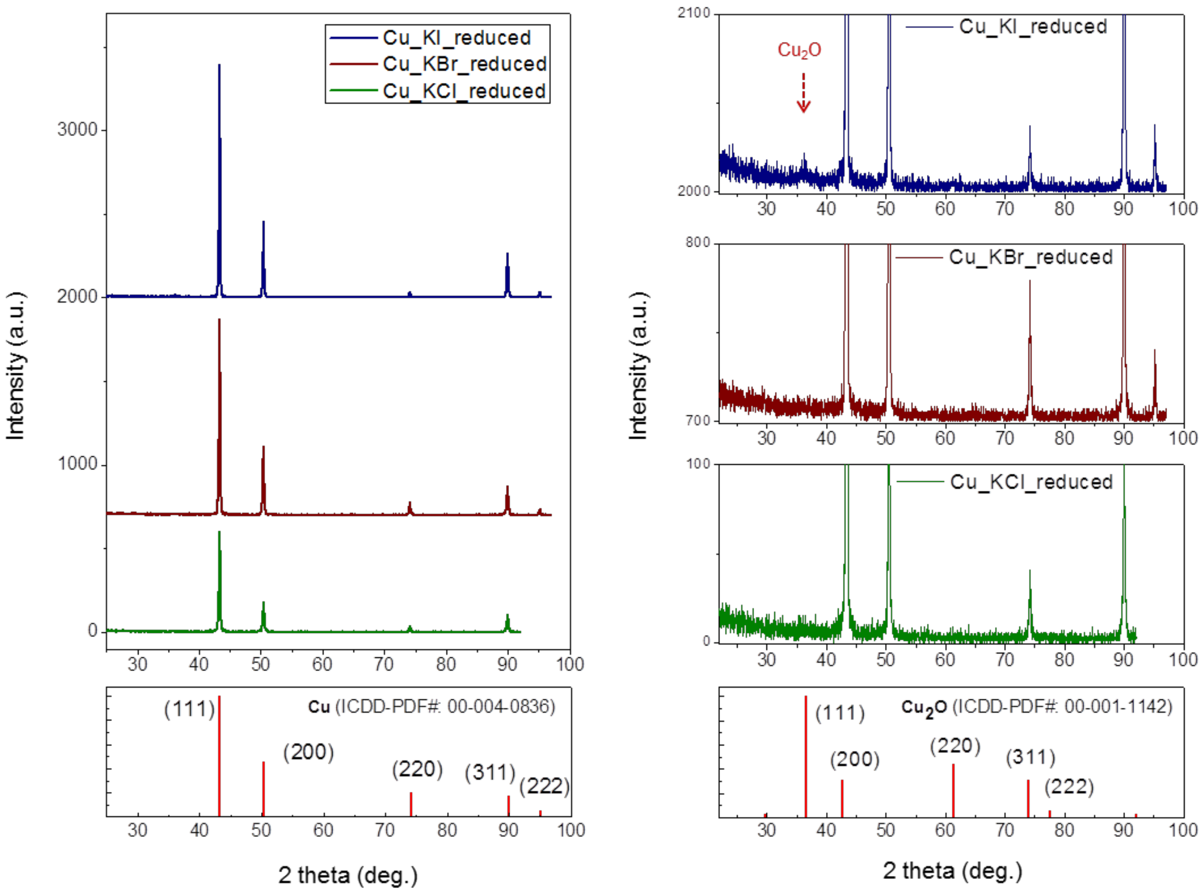

Supplementary Figure 11. GI-XRD data of the Cu_KX samples after immersion in air-saturated $KHCO_3$ and subsequent reduction in CO_2-saturated 0.1 M $KHCO_3$ by LSV from its OCP to -1.8 V at a scan rate of 5 mV/s (left) and the same data magnified (right) to reveal the small peak corresponding to Cu_2O. These GI-XRD data of reduced catalysts (left) are nearly identical to that of the original electropolished Cu shown in Fig. 1a. In the data corresponding to reduced Cu_KI, the peak from Cu_2O (111) appears at 2 theta = 36.6°. This result is consistent with the EDS data (see Fig. 3d and h), which show reduced Cu_KI has a higher content of O relative to Cu_KCl and Cu_KBr. Source data are provided as a Source Data file.

Supplementary Table 1. EDS results of atomic composition and molecular composition of catalysts shown in Fig 4. Results are average values based on three measurements at different locations on the catalyst.

Atomic composition	Electropolished Cu		Cu_KCl			Cu_KBr			Cu_KI		
	Cu	O	Cu	O	Cl	Cu	O	Br	Cu	O	I
As-prepared	98.36	1.64	59.37	2.31	38.32	59.58	2.37	38.06	48.70	16.06	35.20
Immersed	97.22	2.78	71.57	22.57	5.87	65.55	25.47	8.98	49.97	17.69	32.18
Reduced	98.37	1.63	92.56	7.44	0.00	91.70	8.02	0.27	84.35	15.56	0.08

Composition of molecular species	Electropolished Cu		Cu_KCl			Cu_KBr			Cu_KI		
	Cu	Cu_2O	Cu	Cu_2O	CuCl	Cu	Cu_2O	CuBr	Cu	Cu_2O	CuI
As-prepared	98.30	1.70	28.80	4.04	67.15	29.32	4.13	66.55	0.00	16.10	83.90
Immersed	97.06	2.94	41.77	46.21	12.02	13.90	63.68	22.42	0.00	21.66	78.34
Reduced	98.32	1.68	91.25	8.75	0.00	90.09	9.59	0.33	77.20	22.68	0.12

Supplementary Table 2. Conversion of EDS data from atomic composition into molecular composition.

Molecular species	Molecular composition	Atom	Amount of atoms	Normalized atomic Composition	Raw Data
Cu	(1-x-y)	Cu	(1-x-y)+2x+y = 1+x	1+x / (1+2x+y)	0.5937
Cu_2O	x	O	x	x / (1+2x+y)	0.02307
CuCl	y	Cl	y	y / (1+2x+y)	0.3832
Total	1	Total	1+2x+y	1	1

Supplementary Note 1. Conversion of EDS data from atomic composition into molecular composition. For example, in the case of the as-prepared chlorinated Cu (Cu_KCl), in order to convert the atomic composition into the molecular composition, three species are assumed to be present: Cu, Cu_2O, and CuCl. Let x and y represent the molecular composition of Cu_2O and CuCl, respectively. Since the total molecular composition should be 100%, the molecular composition of metallic Cu is then 1-x-y. From the assumed molecular composition, the relative number of atoms can be expressed: atomic Cu comes from metallic Cu (1-x-y), Cu_2O (2x), and CuCl (y). When considering stoichiometry, Cu_2O has two atoms of Cu (2x). Normalizing the atomic composition and solving the simultaneous equations gives the molecular composition: x/(1+2x+y) = 0.02307 and y/(1+2x+y) = 0.3832. Then x = 0.0404, y = 0.6715, (1-x-y) = 0.288.

Supplementary Note 2. Characterization of residual halide with XPS and EDS

Both X-ray photoelectron spectroscopy (XPS) and EDS were used to analyze the catalysts for residual halide after anodic halogenation and subsequent reduction (Supplementary Figures 12 and 13, respectively). Both techniques are used to profile the surface and subsurface of the catalysts to depths up to 10 nm (XPS) or a few microns (EDS).[2,3]

XPS data shown in Supplementary Figure 12 reveals the presence of cuprous halide peaks on the as-prepared Cu_KX samples (at binding energy = 199 eV for Cl $2p_{3/2}$, 68.7 eV for Br $3d_{5/2}$, 619 eV for I $3d_{5/2}$). In contrast, halide peaks are not observed on any of the Cu_KX samples when reduced by LSV from OCP to -1.8 V vs. Ag/AgCl at a scan rate of 5 mV/s.

Similarly, EDS data of Cu_KX samples before and after electroreduction by LSV are shown in Supplementary Figure 13. Only reduced Cu_KI samples (Supplementary Figure 13c) have residual iodide (0.06 atomic% and 0.135 atomic% at 30s and 60s of iodination time), consistent with results shown in Fig. 3 and Supplementary Table 1 (0.08 atomic% at 50s of iodination time). Thus, both XPS and EDS provide strong evidence that only a trace amount of halide remains, and only on the Cu_KI catalyst (up to 0.625 atomic% at 250 s of iodination time). This amount of halide is unlikely to affect electrochemical CO_2 RR based on the following calculation: assume the catalyst has a geometric area of 0.35 cm^2 and a CuI-derived thickness of 2 microns. If all residual iodide ions are released into the electrolyte (8 mL) during electrochemical CO_2 RR, the maximum concentration of iodide is only $7.7*10^{-6}$ M.

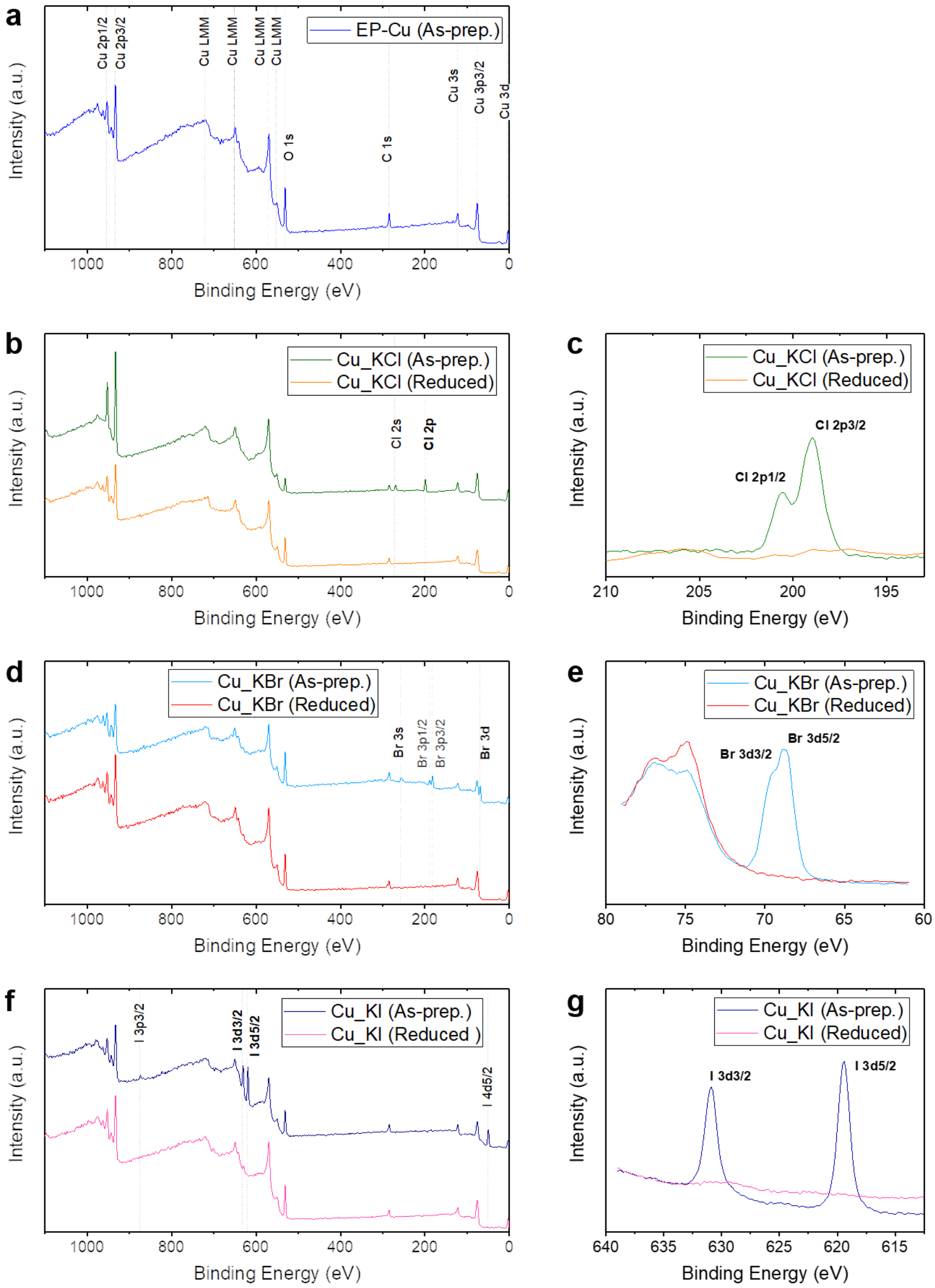

Supplementary Figure 12. X-ray photoelectron spectroscopy (XPS) of (a) electropolished Cu foil (EP-Cu), (b, c) Cu_KCl, (d, e) Cu_KBr, and (f, g) Cu_KI. 'As-prep.' indicates the as-prepared catalyst by electropolishing or anodic halogenation. 'Reduced' indicates the catalyst was reduced in 0.1 M $KHCO_3$ electrolyte by LSV from its corresponding OCP to -1.8 V vs. Ag/AgCl at a scan rate of 5 mV/s. The halogenation time was 100s for Cu_KCl, 100s for Cu_KBr, and 30 s for Cu_KI. Source data are provided as a Source Data file.

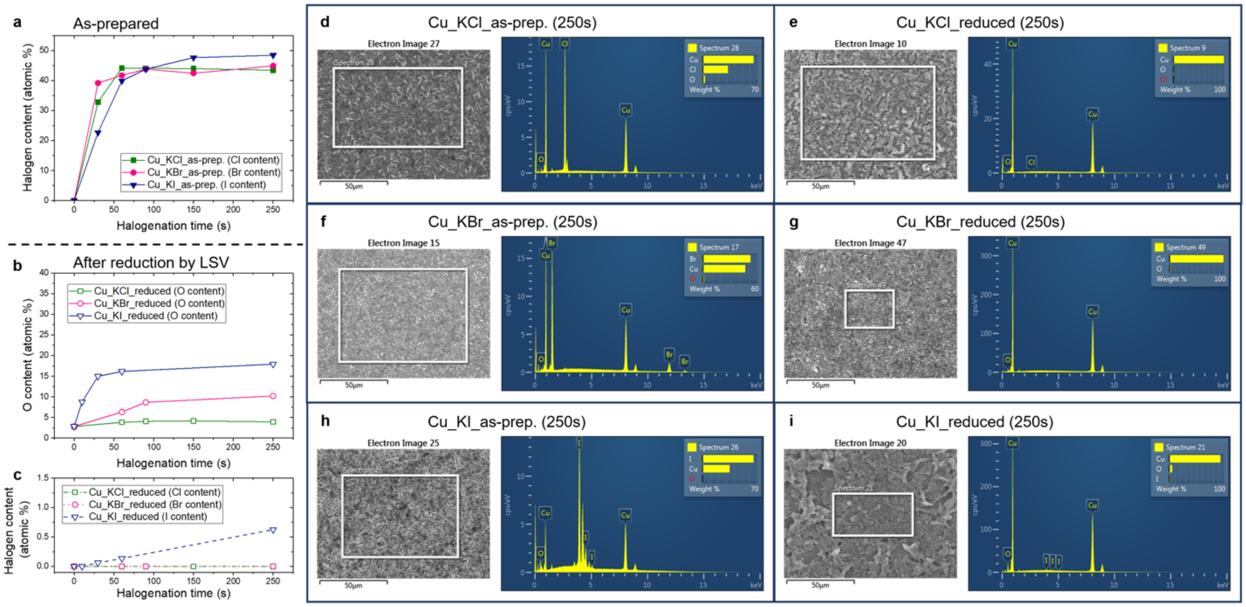

Supplementary Figure 13. Energy-dispersive X-ray spectroscopy (EDS). (a) Halogen content of as-prepared Cu_KX (X = Cl, Br, and I) as a function of halogenation time. (b) Oxygen content of reduced Cu_KX. (c) I content of reduced Cu_KI.SEM images and corresponding raw EDS data of the as-prepared (d, f, h) and the reduced Cu_KX (e, g, i) with halogenation time of 250 s. Source data are provided as a Source Data file.

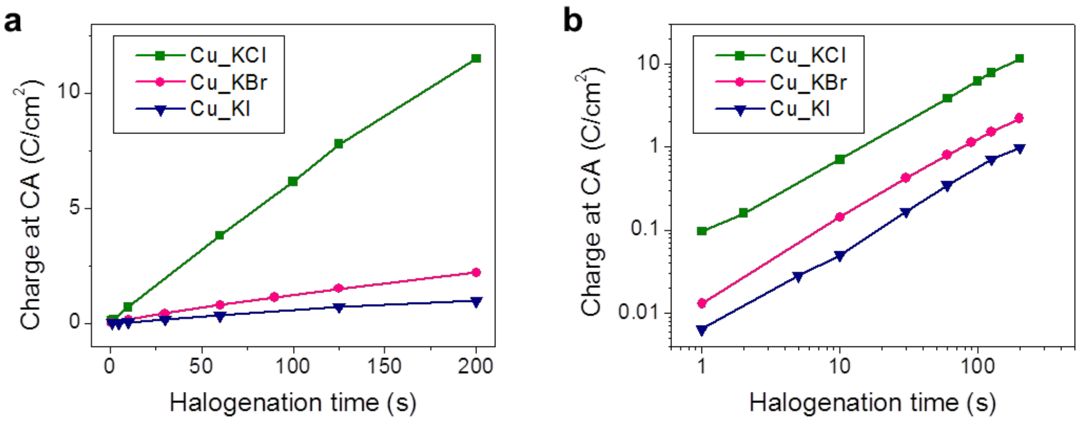

Supplementary Figure 14. Linear scale (a) and log-scale (b) plots of the amount of charge delivered by chronoamperometry (CA) to anodically halogenate Cu foils for different amounts of time. Source data are provided as a Source Data file.

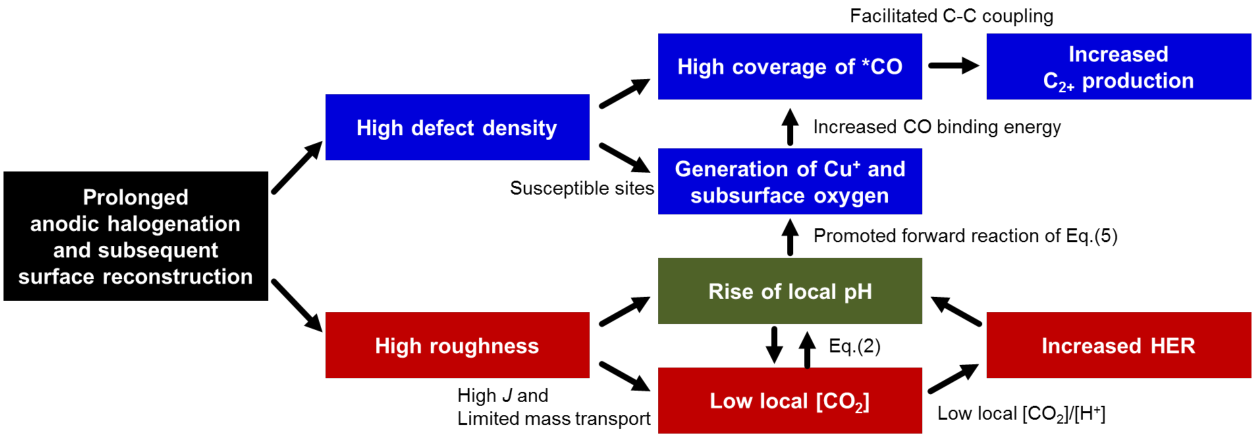

Supplementary Scheme 1. Flow diagram showing relationship of different outcomes caused by excessive anodic halogenation of Cu foils. Merits (blue boxes) and demerits (red boxes) of the impact of prolonged anodic halogenation on the FE of C_{2+} production in electrochemical CO_2 RR. *J* is current density. The rise of local pH shown in the green box is the fulcrum (balance point) between merits and demerits of prolonged anodic halogenation.

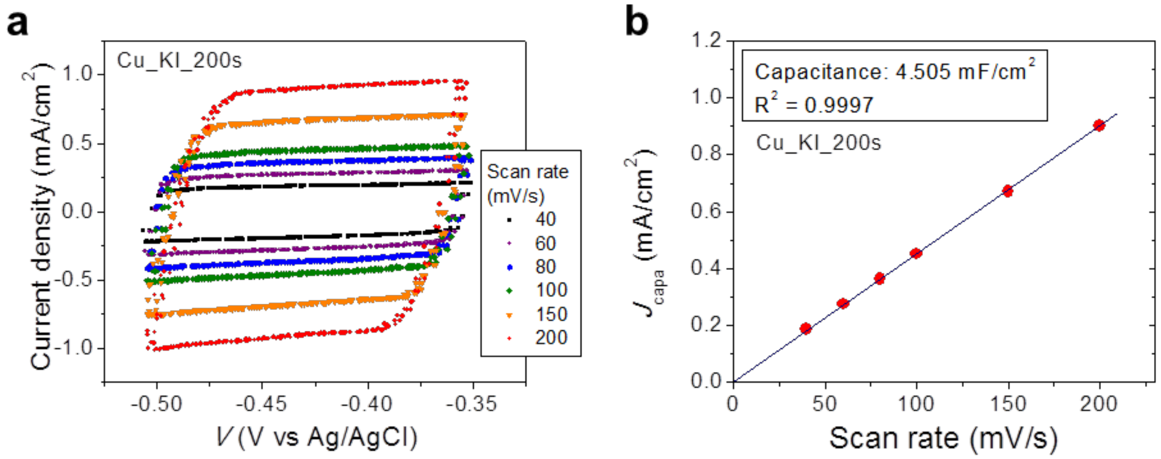

Supplementary Figure 15. Double-layer (DL) capacitance measurements using the method described in Mistry *et al.*[4] (a) Cyclic voltammetry (CV) data of reduced Cu_KI (iodinated in KI for 200 s) at different scan rates between -0.35 V and -0.5 V vs Ag/AgCl, where faradaic processes do not occur in CO_2-saturated 0.1 M $KHCO_3$. (b) A plot of current density collected at -0.425 V vs. Ag/AgCl in CV as a function of the scan rate: $J_{capa} = (J^+ - J^-)/2$, where J^+ is a positive current density at -0.425 V vs. Ag/AgCl and J^- is a negative current density at -0.425 V vs. Ag/AgCl. The slope of the linear regression gives the DL capacitance. Source data are provided as a Source Data file.

Supplementary Note 3. Calculation of concentrations of ions in CO_2-saturated 0.1 M $KHCO_3$ electrolyte.

Concentration of ions were calculated according to the published method by Meenesh R. Singh *et al.*[5]. Dissolution of CO_2 into the electrolyte is in equilibrium given by the Henry's constant (K_0).

$$CO_2\ (g) \leftrightarrow CO_2\ (aq)$$

$$K_0 = C_{CO2} / f_{CO2}$$

where C_{CO2} is the concentration of dissolved CO_2 and f_{CO2} is the fugacity of CO_2 in the gas phase.

Temperature-dependence of K_0 follows the empirical relationship:

$\ln(K_0) = 93.4517(100/T) - 60.2409 + 23.358 \ln(T/100) + S[0.023517 - 0.023656(T/100) + 0.0047036(T/100)^2]$

where T is the temperature in Kelvin and S is salinity in parts per thousand.

Concentrations of dissolved CO_2, bicarbonate (HCO_3^-), and carbonate (CO_3^{2-}) ions are described by the equilibrium equations:

$CO_2\ (aq) + H_2O \leftrightarrow H^+ + HCO_3^-$, $\quad pK_1 = 6.37$, $k_{+1} = 0.0371\ s^{-1}$, $k_{-1} = 86971\ L \cdot s^{-1} mol^{-1}$ (S1)

$HCO_3^- \leftrightarrow H^+ + CO_3^{2-}$, $\quad pK_2 = 10.25$, $k_{+2} = 59.44\ s^{-1}$, $k_{-2} = 1.057 \times 10^{12}\ L \cdot s^{-1} mol^{-1}$ (S2)

where K_1 is the equilibrium constant for reaction (S1), k_{+1} is the forward rate constant of reaction (S1) and k_{-1} is the backward rate constant of reaction (S1), K_2 is the equilibrium constant for reaction (S2), k_{+2} is the forward rate constant of reaction (S2) and $k_{-2}$is the backward rate constant of reaction (S2).

From the reaction (S1),

$$K_1 = [H^+][HCO_3^-]/[CO_2] \tag{S3}$$

From the reaction (S2),

$$K_2 = [H^+][CO_3^{2-}]/[HCO_3^-] \tag{S4}$$

Rearranging equations (S3) and (S4) gives

$$K_1[CO_2]/[H^+] = [H^+][CO_3^{2-}]/K_2 \tag{S5}$$

There is a boundary condition that the initial concentration of $KHCO_3$ is 0.1 M and the added bicarbonate ions can be converted into CO_2 (aq) or carbonate ions:

$$0.1 = [CO_2] + [HCO_3^-] + [CO_3^{2-}] \tag{S6}$$

Then the concentration of the dissolved CO_2 can be expressed as a function of $[H^+]$:

$$[CO_2] = \frac{(0.1)[H^+]}{K_2\left[\frac{K_1}{[H^+]} + \frac{[H^+]}{K_2} + \frac{K_1}{K_2}\right]} \tag{S7}$$

There is an additional generation term for dissolved CO_2 from a constant feed of gaseous CO_2:

$$R_{CO2,feed} = \beta K_0 f_{CO2} \tag{S8}$$

where β is the transfer coefficient ($\beta = k_1 \alpha \delta$), $k_1 \alpha$ is the volumetric mass-transfer coefficient (unit: s^{-1}) on the liquid side of gas–liquid interface, and δ is the fraction of inlet CO_2 transferred from the gas phase to the liquid phase. For this work, $\beta = 0.33\ s^{-1}$ was used, which is the value used in Singh *et al.*[5]

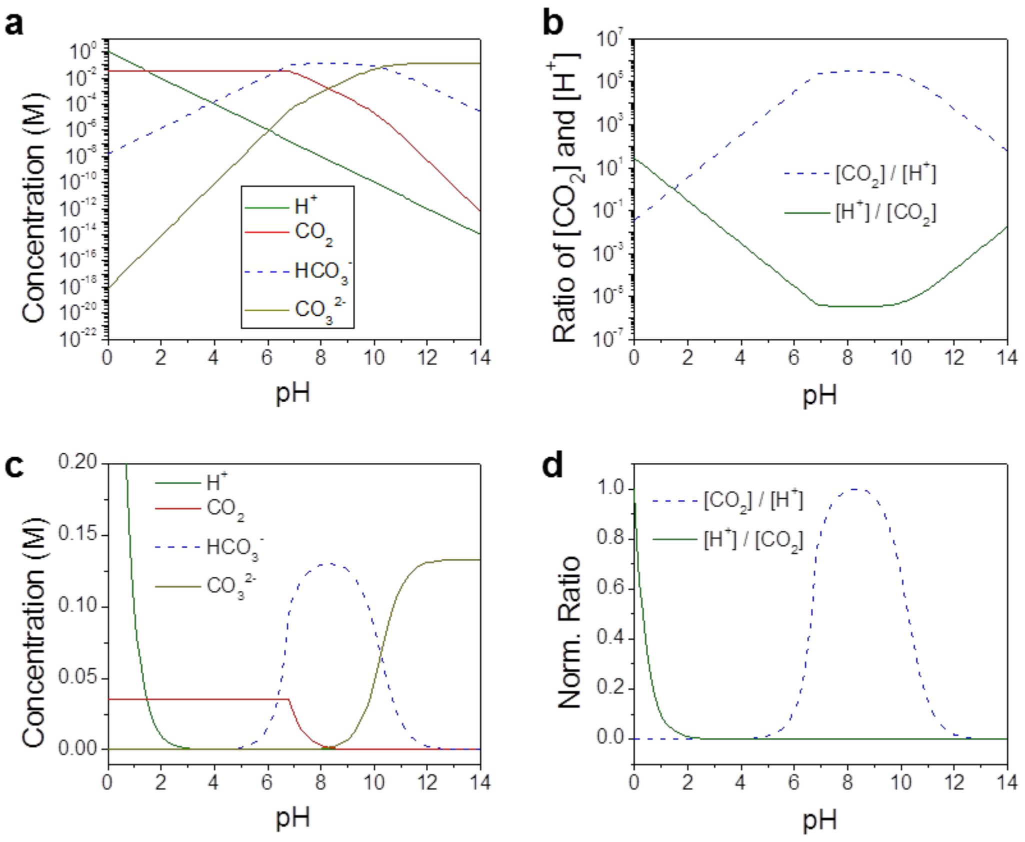

Supplementary Figure 16. Calculated concentration of ions in CO_2-saturated 0.1 M $KHCO_3$ as a function of pH. (a) Log-scale concentration of proton, dissolved CO_2, bicarbonate and carbonate ions. (b) Log-scale concentration ratios of dissolved CO_2 and proton. (c) Linear-scale concentrations of proton, dissolved CO_2, bicarbonate and carbonate ions. (d) Normalized concentration ratios of dissolved CO_2 and protons in linear-scale. (c) and (d) are identical to Fig. 7b and c.

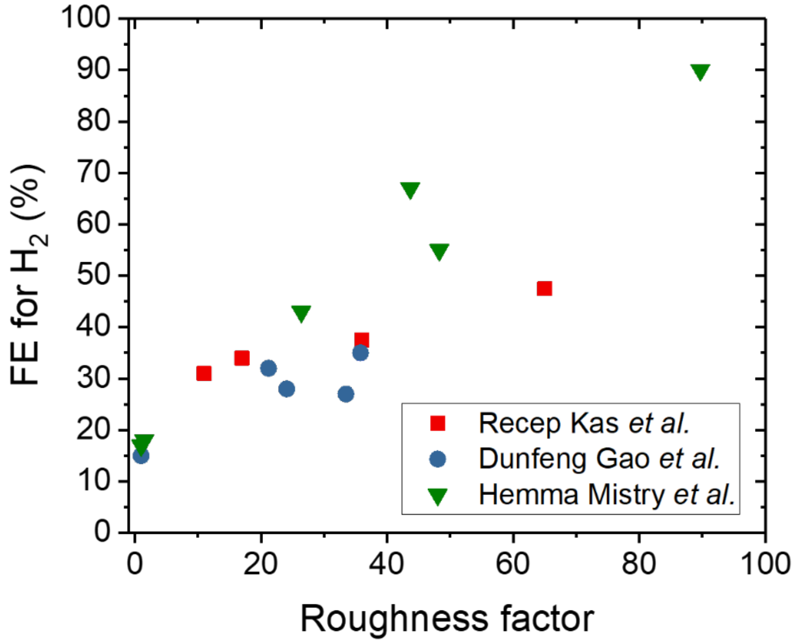

Supplementary Figure 17. FE of H_2 as a function of the roughness factor of Cu foils used to electrochemically reduce CO_2 in CO_2-saturated 0.1 M $KHCO_3$ electrolyte. Data are taken from supplementary references [1,4,8].

Supplementary Table 3. The FE of H_2 and the roughness factor of Cu foils used to electrochemically reduce CO_2 in CO_2-saturated 0.1 M $KHCO_3$ electrolyte. Data are taken from Supplementary Information References [1,4,8].

Authors	Preparation method	Sample	Roughness	FE of H_2 (%)	Overpotential (V vs. RHE)	Ref.
Dunfeng Gao *et al.*	Plasma-activated copper nanocubes	EP-Cu<br>Cube as-prepared<br>Cube O_2 20s<br>Cube Ar 5min<br>Cube H_2 4min	1<br>35.8<br>24.1<br>21.2<br>33.5	15<br>35<br>28<br>32<br>27	-1.02 V	1
Hemma Mistry *et al.*	Plasma-activated copper foil	EP-Cu<br>H_2 100W 2min<br>O_2 20W 2min<br>O_2 100W 2min<br>O_2 100W 2 min + H_2<br>O 100W 10min	1<br>1.5<br>26.4<br>43.7<br>48.3<br>89.7	17<br>18<br>43<br>67<br>55<br>90	-0.975 V	4
Recep Kas *et al.*	Electrodeposition of Cu_2O by reduction of copper(II) lactate	3 C/cm^2<br>5 C/cm^2<br>7 C/cm^2<br>11 C/cm^2	11<br>17<br>36<br>65	31<br>34<br>37.5<br>47.5	-1.1 V	8

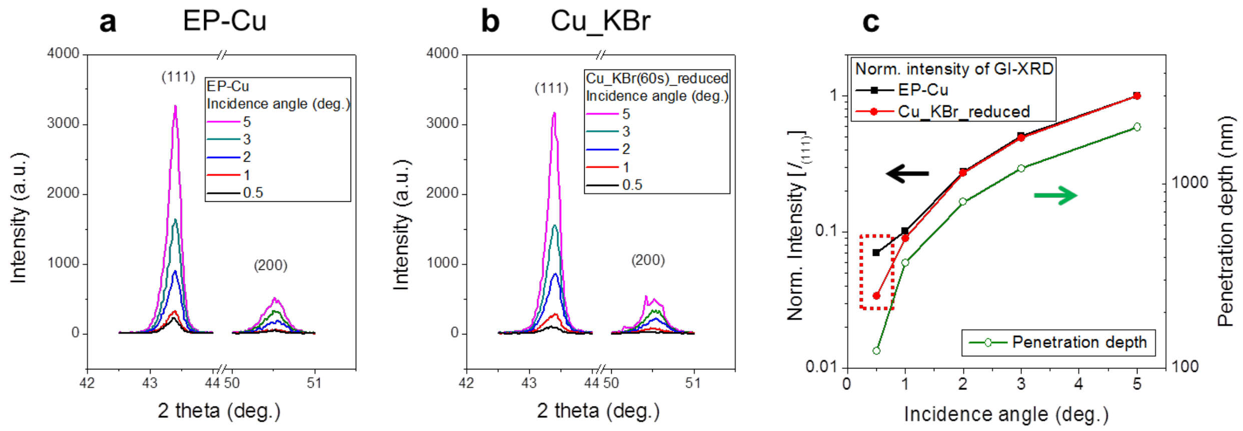

Supplementary Figure 18. GI-XRD data of electropolished Cu (EP-Cu) and Cu-KBr collected at different incidence angles from 0.5° to 5° to show that Cu(I)-halide derived catalysts have a higher density of defect sites than EP-Cu. (a) GI-XRD data of EP-Cu and (b) GI-XRD data of Cu_KBr reduced by LSV. (c) The intensity of the GI-XRD peak at 2theta = 43.3° [corresponding to (111)] in (a) and (b) plotted as a function of incidence angle. The intensity was normalized by the intensity measured at an incidence angle of 5° to eliminate sample-to-sample variations. Because diffraction requires periodic structures, a surface with a high density of defects (i.e., low crystallinity) will exhibit GI-XRD peaks of lower intensity than those of a surface with fewer defects. Thus, the intensity of GI-XRD signal of Cu_KBr decreases relative to that of EP-Cu (highlighted in the dashed red rectangle) due to a high density of defect sites at the surface of Cu_KBr. Also included in (c) is the X-ray penetration depth in Cu plotted as a function of incidence angle. The GI-XRD measurement becomes more surface-sensitive as the incidence angle and corresponding penetration depth decrease (i.e., penetration depth is calculated to be 2025 nm or 123 nm at incidence angle of 5° or 0.5°, respectively). It should be noted that porous or rough surfaces of Cu(I)-halide-derived catalysts may alter refraction at the Cu surface, making *quantitative* determination of defect density with GI-XRD difficult. Source data are provided as a Source Data file.

Supplementary Note 4. Calculation of the penetration depth as a function of incidence angle

The penetration depth as a function of incidence angle was calculated as: [6]

$$z_{1/e} = \frac{\lambda}{4\pi}\sqrt{\frac{2}{\sqrt{(a_i{}^2 - a_c{}^2 + 4\beta^2} - (a_i{}^2 - a_c{}^2)}}$$

where $z_{1/e}$ is the depth at which the intensity is reduced to 1/e, λ is the wavelength of the X-ray, a_i is the incidence angle of the X-ray, a_c is the critical angle of copper, β is the imaginary part of the refractive index of copper or the extinction coefficient. The refractive index can be expressed by $r = 1 - \delta + i\beta$, where δ is the deviation from the real part of the refractive index. Values for δ and β of copper at $\lambda = 1.54$ Å are 2.405×10^{-5} and 5.58×10^{-7}, respectively.[6] The critical angle can be calculated by $a_c = (2\delta)^{0.5}$, which leads to $a_c = 6.935 \times 10^{-3}$ radians = 0.3974° at $\lambda = 1.54$ Å. The critical angle calculated at different wavelengths are given in Supplementary Table 3. The trend of the calculated a_c is consistent with the empirical value of ~ 0.33 ° measured at 9.1 keV.[7]

Supplementary Table 4. Optical constants and the critical angle of copper at x-ray region.

Wavelength (Å)	Photon energy (eV)	δ	β	a_c (rad.)	a_c (°)
1.54	8.055	2.405×10^{-5}	5.58×10^{-7}	0.006935	0.3974
1.392	8.912	1.785×10^{-5}	3.76×10^{-7}	0.005975	0.3423
1.281	9.684	1.672×10^{-5}	23.5×10^{-7}	0.005783	0.3313

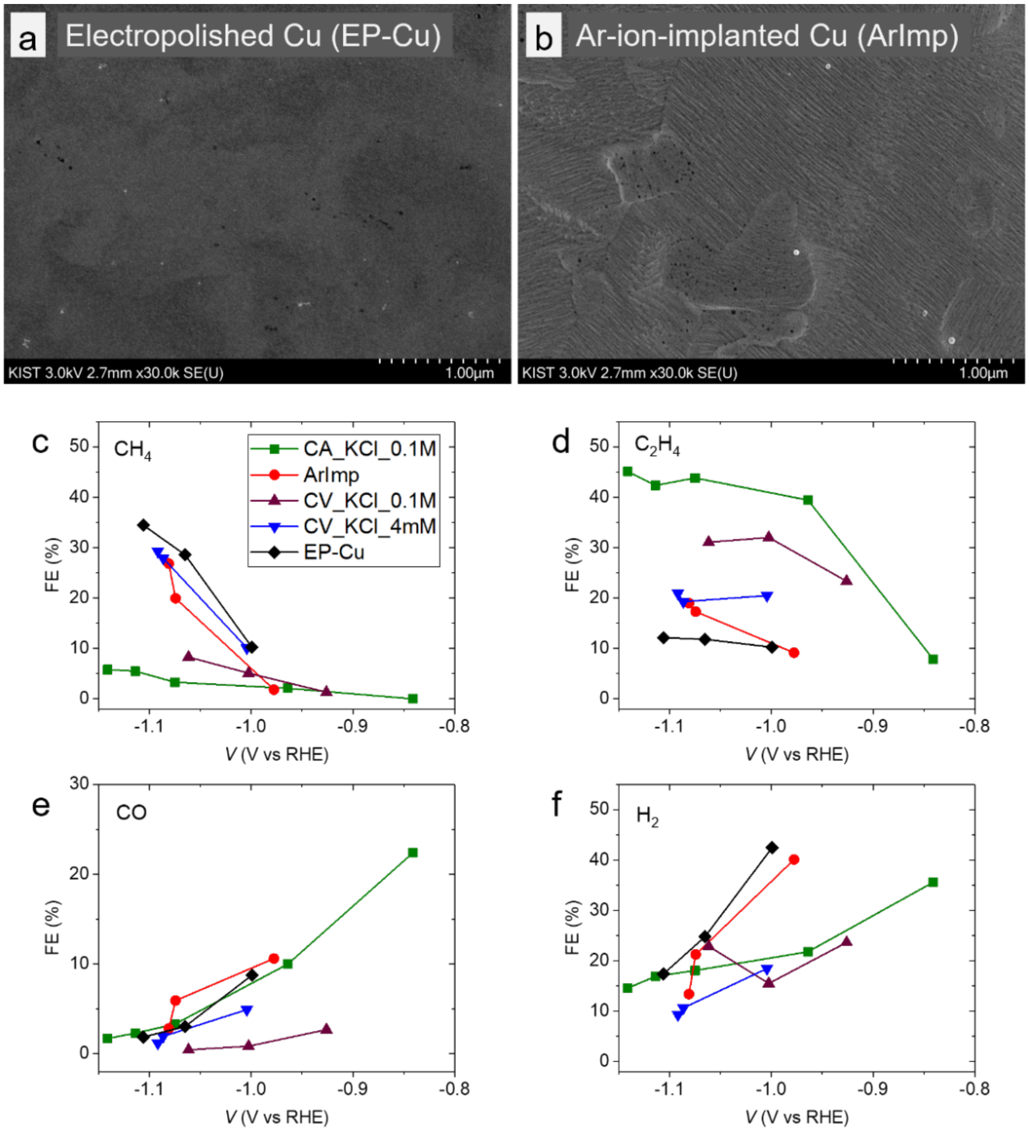

Supplementary Figure 19. Comparison of different methods for generating surface defects on Cu catalysts for electrochemical CO_2 RR. SEM images of (a) electropolished Cu (**EP-Cu**) and (b) Ar-ion-implanted Cu. Plots of FE for (c) CH_4, (d) C_2H_4, (e) CO, and (f) H_2 as a function of applied potential. Legend abbreviations: **CA_KCl_0.1M** corresponds to a Cu catalyst prepared via anodic halogenation (our method) using chronoamperometry (CA) in aqueous 0.1 M KCl at 1.1 V vs. Ag/AgCl; **ArImp** corresponds to a Cu catalyst subjected to Ar-ion-implantation; **CV_KCl_0.1M** corresponds to a Cu catalyst prepared by electrochemical cycling (Ref 41, Chen *et al.*) in aqueous 0.1 M KCl using five triangular potential scans ranging from 0.24 V to 1.74 V vs. RHE at a scan rate of 500 mV/s with the potential held at the positive and negative limits for 10 and 5 seconds, respectively, for each cycle; **CV_KCl_4mM** corresponds to a Cu catalyst prepared by three electrochemical cycles (Ref 14, Roberts *et al.*) between -1.15 and 0.9 V vs. RHE in 4 mM KCl + 0.1 M $KHCO_3$. The FE for C_2H_4 (19.0 % at -1.081 V) is enhanced at **ArImp** compared to **EP-Cu** (12.15 % at -1.106 V). The FE of C_2H_4 at **CV_KCl_4mM** was only 21%, reflecting the low concentration of KCl (4 mM) used in this method and consequential low density of defects generated. The highest FE for C_2H_4 (45%) was observed at **CA_KCl_0.1M**. Source Data are provided as a Source Data file.

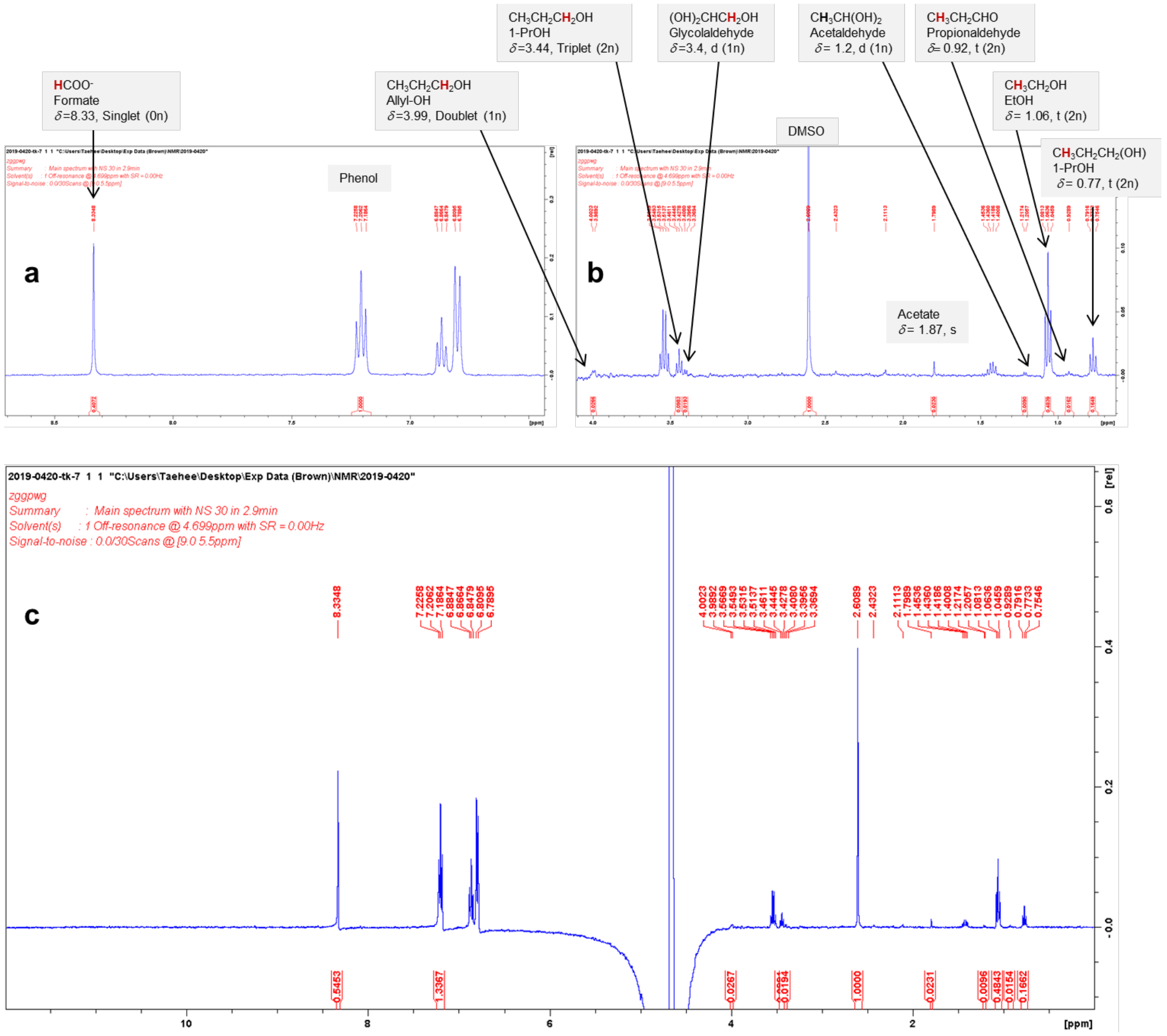

Supplementary Figure 20. NMR analysis of liquid products produced from the electrochemical CO_2 RR. The spectrum shown is typical and corresponds to a reaction catalyzed by Cu_KBr (with anodic bromination time of 200 s). (a) Processed and assigned NMR peaks downfield to the suppressed water peak (chemical shift = 4.7 ppm). (b) Processed and assigned NMR peaks upfield to the suppressed water peak. (c) Raw data.

Supplementary Note 5. Calculation of Faradaic efficiency

Calculation of Faradaic efficiency for gas products:

$$FE_{gas}(\%) = \frac{I}{I_{total}} = \frac{zF\left[\frac{C_{gas}}{10^6}\frac{v/60}{V_m}\right]}{I_{total}} \times 100$$

where I = current used for generating a particular product, mA;

I_{total} = total current used in bulk electrolysis at time when GC sampling was performed, mA;

z = number of electrons transferred to produce a particular product;

F = Faraday's constant, 96485 C/mol;

C_{gas} = volume concentration of gas product as determined by GC, ppm;

v = volume flow rate of CO_2 gas, mL/min;

V_m = molar volume of an ideal gas at 1 atm, L/mol.

Calculation of Faradaic efficiency for liquid products:

$$FE_{liquid}\ (\%) = \frac{Q}{Q_{total}} = \frac{zF\left[C_{liquid}V_{cath}\right]}{Q_{total}} \times 100$$

where Q = charge used for generating a particular product, C;

Q_{total} = total amount of charge flowed in bulk electrolysis for entire reaction time, C;

C_{liquid} = molar concentration of liquid product as determined by NMR, mol/L;

V_{cath} = volume of catholyte, L.

Supplementary Table 5. Faradaic efficiencies of products obtained from electrochemical CO_2 RR vs. applied potential using Cu_KX catalysts shown in Fig 5.

Catalyst	V_{appl} (Ag/AgCl)	J (mA/cm^2)	V_{comp} (Ag/AgCl)	V_{comp} (RHE)	FE (%)												
					CO	CH_4	C_2H_4	H_2	$HCOO^-$	EtOH	PrOH	Other	Total	C1	C2	C3	C2+
EP_Cu	-1.11	0.12	-1.11	-0.509	0	0	0	93	6	0	0	0	99	6	0	0	0
	-1.31	0.39	-1.30	-0.702	0.5	0	0	89.1	9.2	0	0	0	98.9	9.8	0	0	0
	-1.51	1.72	-1.47	-0.871	17.4	0	0	42.9	36.1	0	0	0	96.4	53.5	0	0	0
	-1.71	3.91	-1.61	-1.015	23.4	11.5	12.17	19.4	28.5	1.2	0	0.1	96.2	63.3	13.49	0	13.49
	-1.91	9.23	-1.72	-1.122	10.7	35.7	18.08	10.8	15.9	3	1.6	2.1	97.9	62.3	23.23	1.6	24.84
	-2.11	19.59	-1.78	-1.182	3.6	54.0	13.28	7.2	9.1	4.4	0.7	5.5	97.6	66.6	20.39	3.4	23.82
Cu_KCl (100s)	-1.11	0.92	-1.10	-0.504	11.3	0	0	81.1	6.7	0	0	0	99.1	18	0	0	0
	-1.31	2.62	-1.28	-0.687	28.4	0	0	41.9	28.7	0	0	0	99	57.1	0	0	0
	-1.51	7.15	-1.44	-0.841	22.4	0	7.89	35.6	33.3	0	0	0	99.1	55.6	7.9	0	7.89
	-1.71	16.91	-1.56	-0.964	10.0	2.1	39.45	21.8	17.5	4.2	3.8	0.4	99.3	29.6	44.0	3.8	47.8
	-1.91	31.10	-1.67	-1.075	3.3	3.3	43.85	18.1	14.7	8.0	3.9	3.4	98.6	21.2	52.6	6.6	59.2
	-2.01	36.20	-1.71	-1.114	2.3	5.5	42.35	16.9	13	8.3	4.0	5.8	98.1	20.8	51.9	8.6	60.47
	-2.11	45.45	-1.74	-1.141	1.7	5.8	45.14	14.6	11.2	11.0	3.7	5.7	98.9	18.7	57.7	7.9	65.62
Cu_KBr (60s)	-1.11	0.75	-1.1	-0.503	13.4	0	0	80	3.9	0	0	0	97.3	17.4	0	0	0
	-1.31	2.57	-1.28	-0.678	25.3	0	0	45.4	26.3	0	0	0	97	51.6	0	0	0
	-1.51	6.29	-1.43	-0.827	24.2	0	2.38	35.1	38.1	0	0	0	99.7	62.2	2.4	0	2.38
	-1.71	11.97	-1.57	-0.969	8.4	4.4	27.56	22.4	31.3	2.1	2.5	0.4	99.2	44.1	30.1	2.5	32.65
	-1.91	24.17	-1.64	-1.041	2.5	8.6	46.81	14.9	11.7	8.4	3.6	1.2	97.8	22.9	56.4	3.6	60.07
	-2.01	32.00	-1.71	-1.11	1.6	8.1	51.2	13.6	7.1	11	3.9	1.6	98.1	16.8	63.8	3.9	67.65
	-2.11	42.67	-1.75	-1.151	1.0	9.6	49.47	11.7	5.6	12.8	3.4	6.1	98.2	16.2	65.4	6.3	71.71
Cu_KI (1s)	-1.11	0.87	-1.1	-0.500	5.6	0	0	83.9	8.8	0	0	0	98.3	14.4	0	0	0
	-1.31	3.39	-1.27	-0.674	23.4	0	0	40.8	35.1	0	0	0	99.3	58.5	0	0	0
	-1.51	7.95	-1.42	-0.825	13.3	0	0.88	36	48.9	0	0	0	99.1	62.2	0.9	0	0.88
	-1.71	14.1	-1.57	-0.974	10.6	4.3	25.52	24.2	28.0	3.2	2.7	0.2	98.6	42.9	28.9	2.7	31.59
	-1.91	27.55	-1.66	-1.060	3.0	10.4	42.63	12.6	13.5	8.2	2.7	5.5	98.5	27	52.7	6.3	58.95
	-2.01	35.14	-1.72	-1.125	2.0	11.9	43.18	9.8	10.1	9.6	3.3	8.8	98.6	24	56.0	8.8	64.79
	-2.11	39.96	-1.74	-1.146	2.0	14.0	44.54	8.9	8.7	9.8	3.2	7.7	98.8	24.7	57.4	7.8	65.21

Supplementary Table 6. Faradaic efficiencies of products obtained from electrochemical CO_2 RR using Cu_KX catalysts anodically halogenated for different lengths of time (see Fig 6).

Catalyst	Halog. Time (s)	V_{appl} (Ag/AgCl)	J (mA/cm^2)	V_{comp} (Ag/AgCl)	V_{comp} (RHE)	FE (%)													
						CO	CH_4	C_2H_4	C_2H_6	H_2	$HCOO^-$	EtOH	PrOH	Other	Total	C1	C2	C3	C2+
Cu_KCl	2	-2.11	38.15	-1.77	-1.17	1.9	25.6	40.8	0	5.8	6.0	8.5	1.6	8.7	98.9	33.4	53.5	6.2	59.64
	10	-2.11	43.54	-1.73	-1.13	1.6	10.8	48.3	0	8.7	7.9	11	3.3	7.2	98.7	20.3	61.6	8.1	69.73
	60	-2.11	40.31	-1.69	-1.09	1.6	4.1	50.2	0	12	9.7	10.9	4.3	5.3	98.1	15.4	62.9	7.9	70.73
	100	-2.11	41.54	-1.72	-1.12	1.8	4.9	49	0	14.2	8.4	10.9	4.1	5.1	98.5	15.2	61.3	7.8	69.15
	125	-2.11	40.77	-1.71	-1.11	2.1	3.2	48.9	0	14.6	9.8	10.6	4.7	5.7	99.5	15	61.5	8.4	69.84
	200	-2.11	41.54	-1.66	-1.06	1.7	4.0	48.2	0	16.6	7.6	11.6	4.1	4.7	98.5	13.3	61.7	6.9	68.58
	300	-2.11	51.85	-1.69	-1.09	1.5	4.4	47.8	0	17.4	7.7	11.2	4.2	4.1	98.3	13.6	60.7	6.6	67.28
Cu_KBr	1	-2.11	29.6	-1.76	-1.16	2.3	31.1	32.85	0	7.6	9.0	7.0	1.9	5.4	97.0	42.3	43.7	3.5	47.17
	10	-2.11	38.42	-1.73	-1.13	1.6	12.9	45.31	0	9.3	8.7	10.5	3.4	5.3	97.1	23.2	58.0	6.5	64.56
	30	-2.11	40.0	-1.74	-1.14	1.6	9.6	46.22	0	10.3	9.3	10.3	3.8	7.2	98.5	20.4	58.9	8.8	67.7
	60	-2.11	40.75	-1.66	-1.06	1.8	5.5	48.62	0	12.0	9.6	11.4	3.7	6.5	99.2	16.9	62.2	8.0	70.27
	90	-2.11	43.33	-1.68	-1.08	2.3	1.7	50.94	0	13.8	8.7	12.0	4.4	4.2	98.0	12.6	64.4	7.2	71.54
	125	-2.11	45.33	-1.68	-1.08	1.6	4.2	49.17	0	13.9	9.0	11.9	4.0	4.9	98.7	14.9	62.7	7.2	69.95
	200	-2.11	42.4	-1.75	-1.15	1.7	3.8	49.07	0	15.2	8.4	11.6	3.9	4.7	98.4	13.9	62.2	7.1	69.33
	300	-2.11	41.08	-1.73	-1.13	1.7	5.0	46.10	0	15.1	7.9	12.0	4.0	6.1	98.0	14.7	60.6	7.7	68.24
Cu_KI	1	-2.11	37.6	-1.69	-1.09	2.2	9.6	45.69	0	9.3	12.4	9.0	3.4	6.1	97.7	24.2	56.61	7.6	64.25
	10	-2.11	40.0	-1.69	-1.09	1.5	8.4	49.99	0	9.3	9.2	13.8	5.2	3.6	98.0	19.1	65.3	7.2	72.58
	30	-2.11	43.89	-1.68	-1.08	1.2	4.5	49.57	0	12.3	10.1	13.5	5.3	3.1	97.0	15.8	63.5	7.9	71.4
	60	-2.11	54.86	-1.66	-1.06	1.3	2.5	48.81	0	14.1	12.4	12.7	6.1	3.4	98.5	16.2	62.5	8.4	70.91
	125	-2.11	52.74	-1.64	-1.04	1.6	0.9	48.02	0.5	16.9	9.5	12.4	7.7	1.6	97.4	12.8	60.7	7.3	68.03
	300	-2.11	54.63	-1.69	-1.09	1.4	0.2	45.44	1.2	19.9	9.6	13.2	6.0	1.8	97.7	11.2	59.4	6.8	66.2

Supplementary Note 6. Electrochemical CO_2 RR on a control sample prepared without halide ions

To illustrate the effectiveness of anodic halogenation on electrochemical CO_2 RR catalysis, a control sample of Cu foil oxidized in 0.05 M K_2SO_4 (Cu_K_2SO_4) was used as a catalyst. XRD data of Cu_K_2SO_4 (Fig. 2b) reveals the formation of Cu_2O. SEM images of Cu_K_2SO_4 shown in Supplementary Figure 8 reveals a corresponding surface morphology of octahedrons with predominantly (111) facets. Electrochemical CO_2 RR experiments were done under the same conditions as those using Cu_KX catalysts. A Cu_K_2SO_4 catalyst oxidized for 300 s produced C_2H_4 with a FE of 35.8 % and C_{2+} products with a FE of 47.7 %, which are higher than those obtained using electropolished Cu but lower than those using Cu(I)-halide-derived catalysts. These results show the effectiveness of anodic halogenation in producing catalysts that favor C_{2+} products from the electrochemical CO_2 RR.

Supplementary Table 7. Faradaic efficiencies of products obtained from electrochemical CO_2 RR using Cu catalysts oxidized in 0.05 M K_2SO_4.

Catalyst	Ox. Time (s)	V_{appl} (Ag/AgCl)	J (mA/cm^2)	V_{comp} (Ag/AgCl)	V_{comp} (RHE)	FE (%)												
						CO	CH_4	C_2H_4	H_2	$HCOO^-$	AcO^-	EtOH	PrOH	Total	C1	C2	C3	C2+
Cu_K_2SO_4	100	-2.01	29.0	-1.61	-1.01	3.6	31.7	32.44	10.1	14.06	0.30	4.95	1.34	98.6	49.41	37.69	1.34	39.03
	300	-2.01	34.3	-1.69	-1.09	1.3	27.0	35.85	14.0	8.70	0.21	8.61	3.05	98.8	37.05	44.68	3.05	47.73

Supplementary Table 8. Double-layer (DL) capacitance and roughness of Cu_KX.

Cu_KCl			Cu_KBr			Cu_KI		
Time (s)	Capacitance (mF/cm^2)	Roughness factor	Time (s)	Capacitance (mF/cm^2)	Roughness factor	Time (s)	Capacitance (mF/cm^2)	Roughness factor
1	0.22	1.83	1	0.25	2.12	1	0.76	6.50
2	0.33	2.79	10	0.54	4.62	5	0.74	6.25
10	0.83	7.03	30	0.81	6.86	10	0.77	6.52
60	1.41	11.98	60	0.89	7.60	30	1.31	11.12
100	1.59	13.50	125	1.11	9.45	60	2.01	17.06
125	1.71	14.53	200	1.21	10.26	125	3.60	30.56
200	1.99	16.90				200	4.51	38.28

The capacitance of the electropolished Cu was 0.1177 mF/cm^2. The roughness factor was determined by: roughness factor = (capacitance of a catalyst)/(capacitance of electropolished Cu)

Supplementary References

1. Gao, D. *et al.* Plasma-activated copper nanocube catalysts for efficient carbon dioxide electroreduction to hydrocarbons and alcohols. *ACS Nano* **11**, 4825-4831 (2017).
2. Powell, C. J., Jablonski, A. & Salvat, F. NIST databases with electron elastic-scattering cross sections, inelastic mean free paths, and effective attenuation lengths. *Surface and Interface Analysis: An International Journal devoted to the development and application of techniques for the analysis of surfaces, interfaces and thin films* **37**, 1068-1071 (2005).
3. Prencipe, I., Dellasega, D., Zani, A., Rizzo, D. & Passoni, M. Energy dispersive x-ray spectroscopy for nanostructured thin film density evaluation. *Sci. Technol. Adv. Mater.* **16**, 025007 (2015).
4. Mistry, H. *et al.* Highly selective plasma-activated copper catalysts for carbon dioxide reduction to ethylene. *Nat. Commun.* **7,** 12123 (2016).
5. Singh, M. R., Clark, E. L. & Bell, A. T. Effects of electrolyte, catalyst, and membrane composition and operating conditions on the performance of solar-driven electrochemical reduction of carbon dioxide. *Phys. Chem. Chem. Phys.* **17**, 18924-18936 (2015).
6. Parratt, L. G. Surface studies of solids by total reflection of X-rays. *Phys. Rev.* **95**, 359 (1954).
7. Martens, G. & Rabe, P. The extended X-ray absorption fine structure in the reflectivity at the K edge of Cu. *J. Phys. C: Solid State Phys.* **14**, 1523 (1981).
8. Kas, R. *et al.* Electrochemical CO_2 reduction on Cu_2O-derived copper nanoparticles: controlling the catalytic selectivity of hydrocarbons. *Phys. Chem. Chem. Phys.* **16**, 12194-12201 (2014).
